# Structure‐Based Rational Design of a Selective Hydrolase Inhibitor of the Severe Acute Respiratory Syndrome Coronavirus‐2 Nsp3 Macrodomain

**DOI:** 10.1002/cbic.202500593

**Published:** 2025-11-02

**Authors:** Robin Krishnathas, Konstantin S. Mineev, Nikolaos K. Fourkiotis, Franck Touret, Christos Sideras‐Bisdekis, Aikaterini C. Tsika, Santosh Lakshmi Gande, Verena Linhard, Sridhar Sreeramulu, Frank Lennartz, Manfred S. Weiss, Bruno Coutard, Georgios A. Spyroulias, Harald Schwalbe

**Affiliations:** ^1^ Institute for Organic Chemistry and Chemical Biology Goethe University Frankfurt Max‐von‐Laue‐Strasse 7 60438 Frankfurt am Main Germany; ^2^ Department of Pharmacy University of Patras Patras 26504 Greece; ^3^ Unité des Virus Émergents (UVE) Aix‐Marseille Université IRD 190 Inserm 1207 IRBA Università di Corsica Marseille France; ^4^ Helmholtz‐Zentrum Berlin Macromolecular Crystallography Albert‐Einstein‐Straße 15 12489 Berlin Germany

**Keywords:** GS‐441524 analogs, isothermal titration calorimetry, NMR spectroscopy, phophate bioisoters, severe acute respiratory syndrome, severe acute respiratory syndrome‐coronavirus‐2 Nsp3b

## Abstract

Viral macrodomains, which hydrolyze mono‐ADP‐ribosylated proteins to evade host immunity, represent emerging antiviral targets, yet their druggability remains underexplored. GS‐441524, the active metabolite of remdesivir, has been identified as an inhibitor of the SARS‐CoV‐2 (severe acute respiratory syndrome coronavirus) macrodomain (Nsp3b). Herein, the structure–activity relationship governing macrodomain recognition by the ribosylated moiety using a panel of nucleoside analogs, revealing that phosphate configuration and nucleobase identity critically modulate binding affinity. GS‐441524 derivatives exhibit up to 200‐fold higher affinity compared to adenosine‐based ligands. A novel sulfamoyl derivative demonstrates superior inhibitory potency, attributable to its occupation of the phosphate subsite and formation of a stabilizing hydrogen‐bond network. These findings provide molecular insights into Nsp3b–ligand interactions and establish a rational framework for the development of high‐affinity, structure‐guided inhibitors targeting viral macrodomains.

## Graphical abstract

Structure‐guided design of a sulfamoyl‐modified analog of GS‐441524 yielded a selective, high‐affinity inhibitor of the SARS‐CoV‐2 macrodomain Nsp3b. The compound occupies the phosphate‐binding pocket, forming a stabilizing hydrogen‐bond network that enhances binding affinity (K_D = 0.86 μM). This study establishes sulfamoyl substitution as an effective phosphate bioisostere for rational antiviral macrodomain inhibitor design.

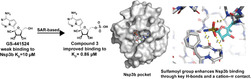

## 1 Introduction

The design and development of low molecular weightcompounds (small molecules) capable of binding and modulating specific biological targets hold immense pharmaceutical relevance—especially in addressing broad‐spectrum viral threats that exploit diverse mechanisms of pathogenesis. Among these, viral macrodomains have emerged as functionally conserved protein domains across several virus families, including Coronaviridae. These macrodomains recognize and hydrolyze mono‐ADP‐ribose (MAR) protein modifications. This biochemical function enables viruses to counteract host immune defenses, which rely on the identification of the protein side chain modification known as ADP‐ribosylation. This modification is a crucial post‐translational alteration involved in immune signaling and stress responses.^[^ ^1^ ^–^ ^3^ ^]^

Despite their biological significance, the therapeutic potential of viral macrodomains remains largely unexplored, and validation through selective chemical probes is still ongoing. Recent functionol studies have demonstrated that the macrodomain mac 1 (Nsp3b) of severe acute respiratory syndrome coronavirus‐2 (SARS‐CoV‐2) is not only conserved but also essential for viral pathogenesis and immune modulation. Even in the absence of replication defects, highlighting its potential as an antiviral target.^[^ ^4^ ^,^ ^5^ ^]^ A recent review by O’Connor et al. further emphasizes the pressing need for selective, structurally validated chemical ligands to probe Nsp3b biology and guide future drug discovery.^[^ ^6^ ^]^

A promising avenue for modulating Nsp3b function involves repurposing or modifying known antiviral scaffolds. GS‐441524, the nucleoside parent of remdesivir, has demonstrated potent activity against multiple RNA viruses including SARS‐CoV‐2 and feline infectious peritonitis virus (FIPV)^[^ ^7^, ^8^ ^–^ ^9^ ^]^ as transcription inhibitors. Structurally analogous to adenosine, GS‐441524 is incorporated into viral RNA during transcription relying on viral RNA‐dependent RNA polymerases, thereby inducing premature chain termination. Interestingly, prior work from our group showed that GS‐441524 also binds to viral macrodomains, functioning as a hydrolase inhibitor.^[^ ^10^ ^]^ Building upon this observation, our current study expands both chemically and mechanistically to optimize ligand design and probe binding determinants.

To guide rational inhibitor design against this evasion of the immune response, a critical first step involves elucidating the molecular rules that govern ligand recognition by macrodomains, particularly the viral mac1 of SARS‐CoV‐2. In this context, we explored how variations in nucleobase identity and phosphate architecture influence macrodomain‐ligand interactions using several viral or human macrodomains and a panel of adenosine analogs and GS‐441524 phosphate derivatives. Therefore, we systematically dissected how these chemical features shape affinity and selectivity, providing a structural and energetic framework for next‐generation inhibitor development.

Bioisosteric replacement of metabolically labile or charged functional groups is a foundational strategy in medicinal chemistry to enhance drug‐like properties while preserving key molecular interactions. Phosphate groups, though critical for binding in many biological systems, often suffer from poor cell permeability, high polarity, and susceptibility to enzymatic hydrolysis, limiting their pharmacokinetic profile. In this context, sulfamoyl groups (–SO_2_NH_2_) have emerged as neutral or weakly basic phosphate bioisosteres, capable of mimicking the geometry and hydrogen‐bonding capacity of phosphate while improving membrane permeability, metabolic stability, and chemical tractability.^[^ ^11^ ^]^ The incorporation of sulfamoyl moieties thus offers a rational avenue for enhancing ligand potency and drug‐likeness, particularly in the context of nucleotide‐inspired inhibitor design.^[^ ^11^ ^,^ ^12^ ^]^ In addition to structure–activity relationships (SAR), we synthesized a novel sulfamoyl‐modified derivative of GS‐441524, hypothesizing that its altered phosphate mimic could enhance interaction with the Nsp3b binding pocket. We subsequently evaluated binding affinity using isothermal titration calorimetry (ITC) and detailed the protein‐ligand architecture via high‐resolution NMR spectroscopy. These integrative efforts revealed that the sulfonamide moiety occupies the subsite of the Nsp3b that binds to the ribosyl‐phosphate groups, forming a stabilizing hydrogen‐bond network with key residues and significantly improving de‐mono‐ADP‐ribosylation (de‐MARylation) inhibition.

Taken together, this study combines chemical synthesis, biophysical profiling, and structural analysis to 1) clarify the molecular determinants of Nsp3b recognition and 2) lay the groundwork for developing potent, selective small‐molecule inhibitors. By leveraging both natural nucleoside analogs and synthesized derivatives, we aim to establish a critical foundation for the rational design of advanced tool compounds and therapeutic agents aimed to counter viral infections, particularly those that exploit Nsp3b‐mediated immune evasion mechanisms.

## 2 Results and Discussion

### 2.1 Phosphate‐Dependent Ligand Recognition by the Nsp3b Highlights the Central Role of Nucleobase Identity

Recent evidence from our group established that GS‐441524—the parent nucleoside of remdesivir—binds to the Nsp3b with an affinity comparable to that of the endogenous metabolite ADP‐ribose (ADPr).^[^ ^10^ ^]^ Given the therapeutic and mechanistic relevance of this interaction, we sought to systematically dissect how specific chemical features of adenosine, and GS‐441524 derivatives contribute to the molecular recognition to Nsp3b, with particular focus on the role of phosphate substitution.

**Figure 1:**
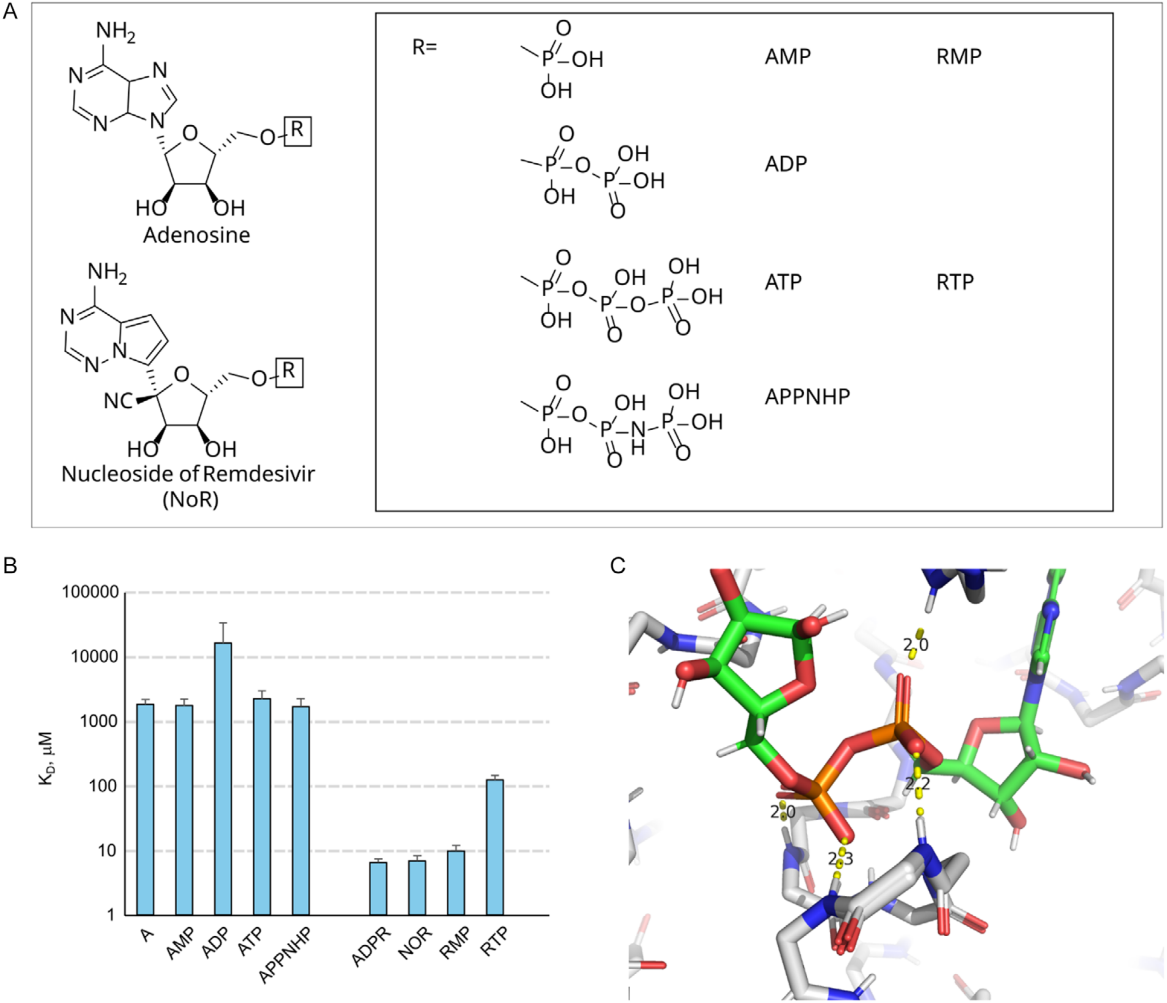
Screening of phosphorylated derivatives of adenosine and GS‐441524 as Nsp3b binders. A) Structures of the compounds tested in this study. B) Affinities of the compounds obtained in the fast screening, error bars represent the standard deviation of the mean. C) Spatial structure of ADPr in complex with Nsp3b of SARS‐CoV‐2 (7TWX 10) with the indication of hydrogen bonds formed by the phosphate groups.

To this end, we performed a SAR analysis encompassing seven nucleotides (Figure S1, Supporting Information) and nucleotide analogs: adenosine, AMP, ADP, ATP, the non‐hydrolyzable ATP analog APPNHP, and two phosphorylated derivatives of GS‐441524—its monophosphate (RMP) and triphosphate (RTP) forms (**Figure** **1**A). These compounds span a continuum from minimal ligand scaffolds to ADPr‐like structures, allowing us to parse how nucleobase identity and phosphate identity (mono‐, di‐, or triphosphate) modulate Nsp3b engagement. Notably, RMP and RTP structurally recapitulate ADPr's extended phosphate‐ribose scaffold while preserving the modified nucleobase of GS‐441524. RMP was synthesized via an in‐house route (Figure S2 & S3, Supporting Information).

We employed a streamlined ^1^H‐^15^N HSQC‐based NMR titration approach, wherein 100 μM Nsp3b was titrated with each ligand at 2x, and 10x molar equivalents. Chemical shift perturbations (CSPs) were quantified and interpreted to estimate binding affinities in the low micromolar to millimolar range (Figure S1, Supporting Information). Full titration curves (≥10 concentrations) were acquired for ligands exhibiting CSPs consistent with high‐affinity binding (Figure 1B).

The resulting affinity profiles revealed two striking trends. First, adenosine and its phosphate derivatives—including AMP, ATP, and APPNHP—exhibited uniformly weak binding (KD ≈ 1.9–2.5 mM), with ADP binding an order of magnitude even more weakly (KD > 10 mM), despite its structural proximity to ADPr. These data implicate the terminal ribose of ADPr as a crucial determinant of high‐affinity recognition by Nsp3b—its absence in ADP severely compromises binding.

Second, GS‐441524 derivatives retained robust affinity, with RMP and RTP exhibiting KD values of ≈10 μM and ≈100 μM, respectively—up to 200‐fold stronger than their adenosine‐based counterparts. This marked enhancement suggests that substitution of adenosine with the C‐linked GS‐441524 base contributes ≈2.5–3.0 kcal mol^−1^ in favorable binding free energy. Given the spatial constraints of the Nsp3b pocket, this energy gain is consistent with the formation of 1–2 additional hydrogen bonds or enhanced stacking interactions, in line with structural models of the Nsp3b–ligand complex.

Together, these findings reveal the dual importance of nucleobase composition and phosphate architecture in Nsp3b recognition. The enhanced binding of GS‐441524 derivatives, despite lacking ADPr's distal ribose, underscores the privileged role of non‐natural nucleobases in reprogramming viral protein interactions—highlighting a key molecular principle exploitable for Nsp3b‐targeted antiviral design.

### 2.2 Phosphate Substitution and the Rationale for Sulfamoyl Modification

The observed influence of phosphorylation on GS‐441524 affinity toward the Nsp3b is unexpectedly modest, and in some cases, counterintuitive. Based on cocrystal structures of Nsp3b with ADPr and AMP^[^ ^10^ ^,^ ^13^ ^]^ one would anticipate that phosphorylated derivatives would stabilize ligand binding via extensive hydrogen bonding within the phosphate‐binding subsite (Figure 1C). Contrary to this expectation, we found that the monophosphate derivative of GS‐441524 (RMP) displayed reduced affinity relative to the parent nucleoside. Similarly, ADP, the diphosphate derivative of adenosine, also bound weakly despite the fact that in the ADPr–Nsp3b complex, each phosphate forms multiple stabilizing hydrogen bonds.

These findings suggest that phosphate groups, although capable of forming hydrogen bonds, may also introduce unfavorable electrostatic and steric effects in the Nsp3b binding pocket. Specifically, the high negative charge density and uncoordinated electronegative oxygen atoms may contribute to repulsive interactions or disrupt optimal ligand orientation. Furthermore, phosphates are prone to enzymatic hydrolysis, limiting the metabolic stability and bioavailability of phosphate‐containing compounds.

To circumvent these limitations, we pursued the incorporation of a sulfamoyl moiety (‐SO_2_NH_2_) at the 5^′^‐position of the GS‐441524 scaffold. The sulfamoyl group is an isosteric and electronic analog of phosphate, yet it offers distinct advantages: it is either neutral or positively charged, provides an additional hydrogen bond donor, and is more resistant to enzymatic degradation. These features enhance pharmacokinetic properties, including membrane permeability and metabolic stability, making sulfamoyl substitution an attractive strategy for ligand optimization.^[^ ^11^ ^,^ ^12^ ^]^

**Figure 2:**
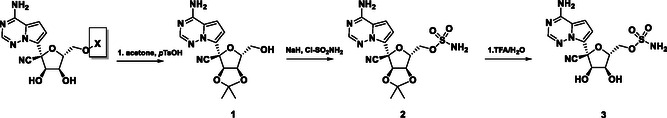
Synthesis of compound **3**. Acetal formation of the starting material with acetone and p‐TsOH affords 1. Treatment of 1 with NaH and sulfamoyl chloride yields 2, which upon deprotection with TFA/H_2_O provides 3.

We synthesized the sulfamoyl derivative (compound **3**) via a concise, three‐step route (**Figure** **2**). The 2′‐ and 3′‐hydroxyl groups of GS‐441524 were protected via acetal formation using acetone under acidic conditions, resulting in compound 1 (Figures S4, S5, Supporting Information).^[^ ^14^ ^,^ ^15^ ^]^ Subsequent selective functionalization of the free 5′‐hydroxyl was achieved by nucleophilic substitution with sulfamoyl chloride in the presence of NaH, yielding the 5′‐O‐sulfamate intermediate 2 in 72% yield (Figures S6, S7, Supporting Information). Finally, acidic deprotection using trifluoroacetic acid (TFA/H_2_O) afforded compound **3** in 84% yield (Figures S8, S9, Supporting Information).

### 2.3 Compound 3 Exhibits Submicromolar Affinity for the Nsp3b

To characterize the molecular interaction between compound **3** and the Nsp3b, we employed a combination of solution‐state NMR spectroscopy and ITC to define its binding affinity and thermodynamic profile.

**Figure 3:**
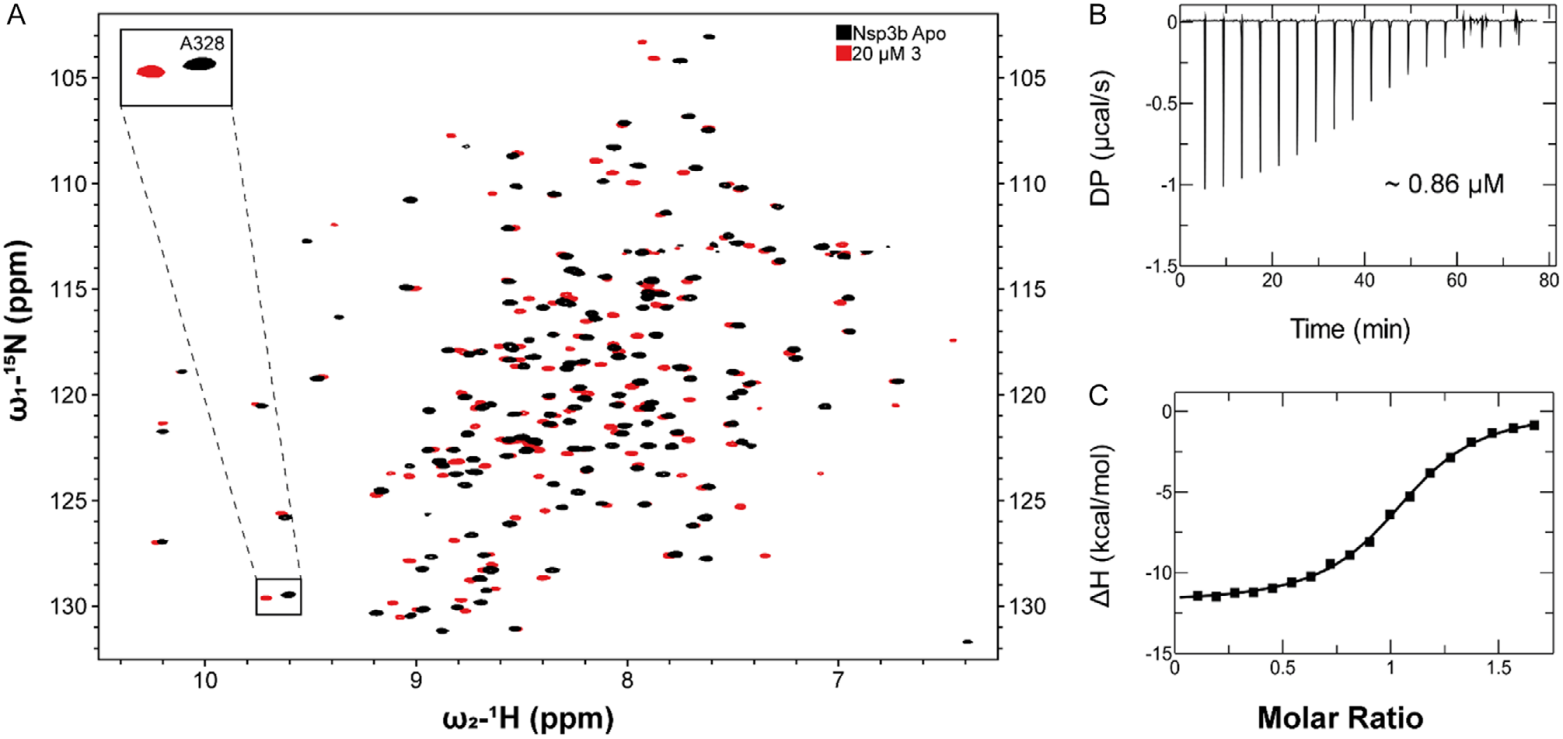
Binding of compound 3 to Nsp3b investigated by NMR and ITC. A) [^1^H, ^15^N] TROSY NMR spectra of Nsp3b in the apo state (black) and in complex with compound 3 at a 1:1 molar ratio (red), recorded at 298 K, concentration of protein was 20 µM, B) ITC measurements of compound 3 binding to Nsp3b. The upper plot B) displays raw ITC data, showing heat changes upon ligand injection, while the lower plot in the panel C) represents the integrated binding enthalpy (ΔH) as a function of the ligand‐to‐protein molar ratio. The experimental points were approximated with the theoretical dependence (solid line), providing the KD value of 0.86 µM. ITC experiments were run at the protein concentration of 26.1 μM.

^1^H‐^15^N HSQC‐based titration experiments revealed significant chemical shift perturbations upon incremental addition of compound **3** to isotopically labeled Nsp3b. The emergence of new, well‐dispersed resonances corresponding to the ligand‐bound form indicated slow exchange kinetics on the NMR timescale, consistent with a high‐affinity interaction (**Figure** **3**A). Resonance assignments for the bound state were performed and are presented in Supporting Figure S10.

Given the slow exchange behavior and the high affinity of the interaction, accurate determination of *K*_D_ and other binding parameters, were obtaiend by ITC measurements under optimized conditions (detailed description of the ITC experiments follows). The resulting isotherm yielded a *K*_D_ of 0.86 ± 0.04 µM (Figure 3B,C), representing an order‐of‐magnitude improvement relative to GS‐441524 monophosphate (RMP) and ADPr.^[^ ^10^ ^]^

Thermodynamic parameters indicated that binding is predominantly enthalpy‐driven, with a measured enthalpy change of ΔH = −12.00 ± 0.07 kcal mol^−1^. This suggests that specific hydrogen bonding and favorable electrostatic interactions are the principal contributors to complex stabilization. Collectively, these data demonstrate that sulfamoyl substitution at the 5′‐position of GS‐441524 confers a substantial gain in binding affinity and thermodynamic favorability, validating this neutral phosphate isostere as a strategic scaffold modification for Nsp3b‐targeted ligand development.

### 2.4 Hybrid NMR/X‐Ray Spatial Structure of Compound 3•Nsp3b Complex Reveals Additional Hydrogen Bonding via the Sulfamoyl Moiety

Having established submicromolar binding affinity of compound **3** to Nsp3b via NMR titration and ITC, we next sought to elucidate the structural determinants underlying this interaction.

Initial attempts to obtain high‐resolution cocrystal structures by soaking or cocrystallization were unsuccessful. While conformational incompatibilities or steric hindrance might play a role, it is also possible that other factors, such as crystal packing effects or the intrinsic dynamics of the binding pocket, contributed to the lack of crystallization.

To overcome this limitation, we employed a hybrid structural modeling approach integrating high‐resolution NMR data with available X‐ray crystal structures of Nsp3b. Given the high spectral quality and resolution of the NMR data (Figure S10, Supporting Information), we pursued solution‐state structural elucidation collecting intermolecular nuclear Overhauser effect (NOE)‐based distance restraints (Figure S11, Supporting Information). Recognizing the high structural conservation among ligand‐bound conformations of Nsp3b, we employed a strategy analogous to NMR^2^ molecular replacement.^[^ ^16^ ^]^ This approach utilized a rigid or semi‐rigid protein template from high‐resolution X‐ray structures, supplemented with experimental NOEs from ^13^C, ^15^N‐filtered NOESY experiments. Using the X‐filtered NOESY experiment, we identified 19 distinct intermolecular distance restraints (Figure S11, Supporting Information), which were subsequently employed to guide the docking of the ligand into the protein conformation derived from the X‐ray crystal structure of the Nsp3b•AMP complex (PDB ID: 6W6Y; resolution: 1.45 Å).^[^ ^10^ ^]^To evaluate alternative structural templates, we also considered four additional high‐resolution X‐ray structures of the Nsp3b: the apo form (0.95 Å), and its complexes with GS‐441524 (1.75 Å), ADP‐ribose (ADPr; 0.90 Å), and ADP‐ribose phosphate (ADPRP; 2.05 Å).^[^ ^10^ ^,^ ^13^ ^,^ ^17^ ^]^ Among these, 6W6Y yielded the lowest number of NOE restraint violations (Table S1, Supporting Information), and exhibited the best agreement with experimentally determined ^*3*^ *J*_NH, H*α*_ coupling constants, particularly in regions predicted to engage with the sulfamoyl moiety of compound **3** (Figure S12, Supporting Information). Additionally, this template provided a favorable hydrogen binding network between the ligand and the protein (Figure S13, Supporting Information), while offering the best overall fit to the NMR data.

### 2.5 Solution‐State NMR Reveals Key Interactions Between Compound 3 and Nsp3b

**Figure 4:**
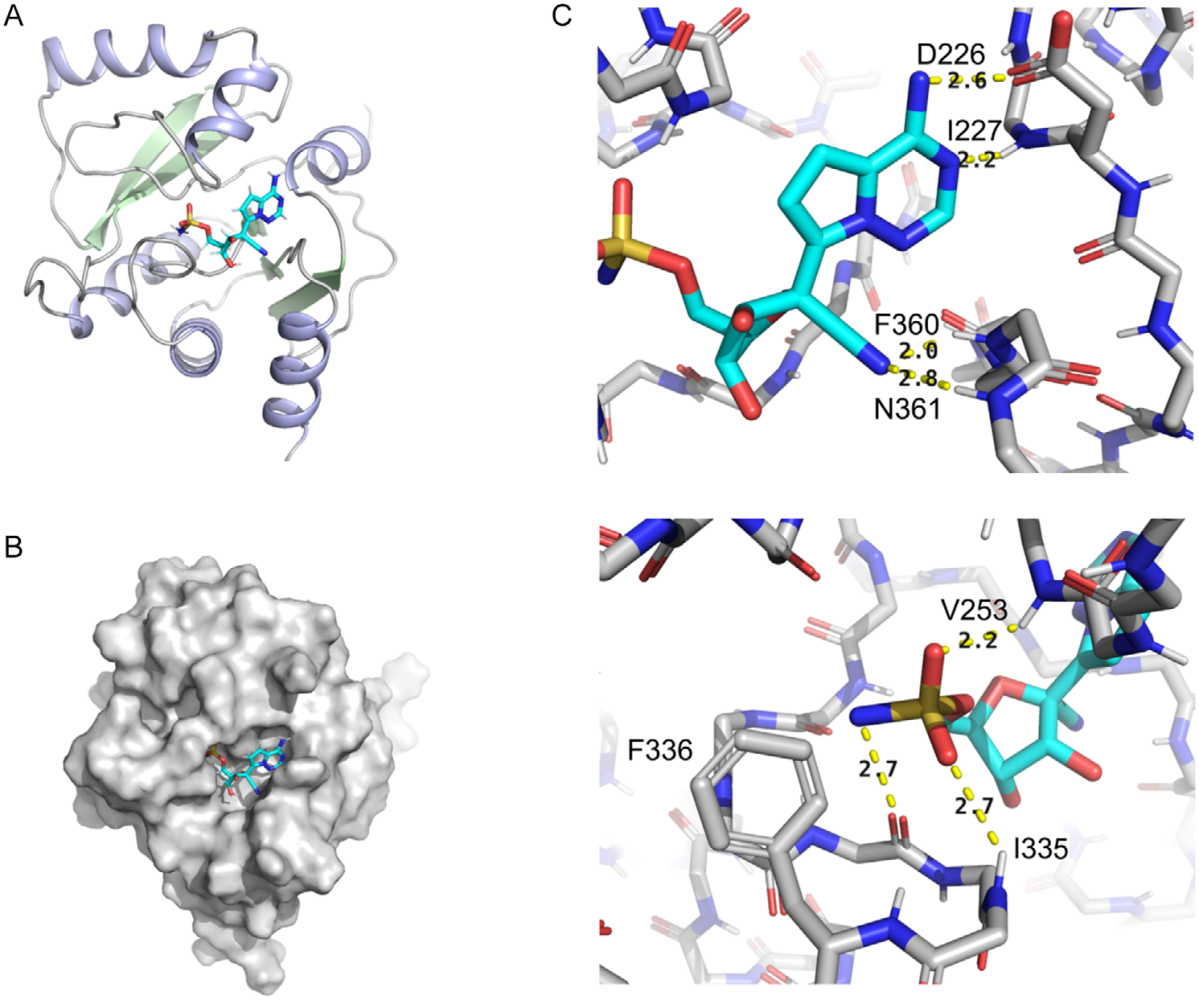
Spatial structure compound 3•Nsp3b. A) 3D structure of 3/Nsp3b complex is shown in ribbon representation, ligand is shown in cyan sticks. B) The ligand is shown on the surface of Nsp3b. C) Key intermolecular interactions, observed in the complex, are shown by yellow dashed lines, the corresponding distances are indicated in angstroms.

The resulting complex structure (**Figure** **4**) revealed a canonical binding mode of the GS‐441524 core, with the nucleobase stabilized by three key hydrogen bonds: 1) N1 to the backbone amide of Ile227, 2) the 6‐amino group to the side chain of Asp226, and 3) the 1′‐cyano group to the amide of Phe360. Additional stabilization is likely contributed by Asp361, whose backbone amide lies proximal to the cyano group. The sulfamoyl group of compound **3** is positioned within the conserved phosphate‐binding subsite, occupying a region analogous to that engaged by AMP and ADPr. Although its exact orientation could not be definitively resolved by NMR spectroscopy due to inherent conformational flexibility, rotameric analysis suggested a single predominant configuration capable of forming a stabilizing triad of hydrogen bonds. In this configuration, the sulfamoyl oxygen atoms accept hydrogen bonds from the backbone amide protons of Val253 and Ile335, while the sulfonamide nitrogen donates a hydrogen bond to the carbonyl oxygen of Gly334. Supporting this model, Val253 exhibited pronounced chemical shift perturbations upon ligand binding (Figure S14, Supporting Information), consistent with direct interaction at the binding interface.

Furthermore, if protonated under physiological conditions, the sulfonamide ‐NH_3_^+^ moiety of compound **3** may engage in a cation–*π* interaction with the aromatic side chain of Phe336, potentially contributing an additional stabilizing effect to the ligand–protein complex. To experimentally evaluate the likelihood of this protonation state, we determined the pK_a_ of the sulfamoyl group by monitoring the chemical shift of the 5′‐CH_2_ proton during pH titration (Figure S15, Supporting Information). The analysis yielded a pK_a_ of 9.37 ± 0.02, indicating that partial protonation of the sulfonamide group is feasible at physiological pH, thereby supporting the potential for cation–*π* interactions in the bound state.

Overall, the proposed structural model is consistent with the enthalpy‐driven binding signature observed in ITC, characterized by a favorable enthalpic contribution (ΔH = −12.00 kcal mol^–^1; –6.75 kcal mol^−1^ compared to GS‐441524). This thermodynamic profile supports the formation of multiple specific and energetically favorable interactions between the sulfamoyl group and the Nsp3b binding pocket, including hydrogen bonds and a potential cation–*π* interaction. Collectively, these findings support the sulfamoyl group as an effective phosphate bioisostere capable of mimicking key polar contacts while enhancing binding affinity through additional interactions. While further biological validation is needed, this study provides a structural rationale for the incorporation of sulfamoyl substitutions in the design of high‐affinity ligands targeting viral macrodomains.

### 2.6 Inhibition of Nsp3b by Compound 3 Assessed via de‐MARylation Assays

**Figure 5:**
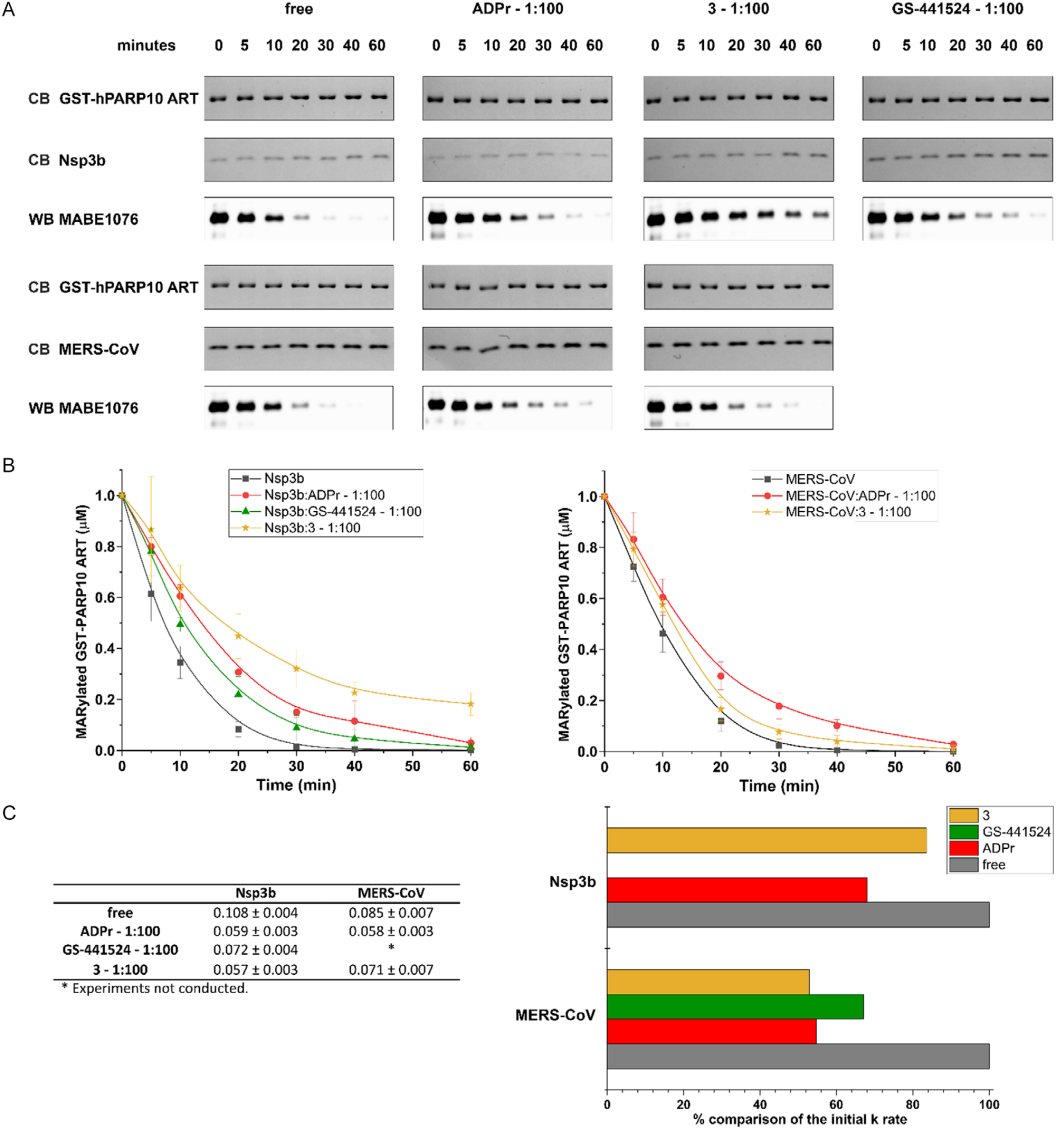
Inhibition of de‐MARylation enzymatic activity by GS‐441524 and compound 3. A) The de‐MARylation activity of Nsp3b and MERS‐CoV macrodomain assessed using immunoblotting. Macrodomains incubated with MARylated GST‐hPARP10 ART at a molar ratio of 1:1, both in the absence and presence of each mentioned compound and molar ratio. Samples were collected at the specified intervals. The total protein amount was verified using Coomassie Blue (CB) stain. The experiments were conducted in triplicate. B) Results of the quantification for the experiments depicted in panel (A) for Nsp3b (left) and MERS‐CoV macrodomain (right). The bands from each experiment were quantified using Image Lab software. Plots and initial substrate decay rate constant (k) calculations were derived using Origin2019b, summarizing the results from each independent experiment for the compounds tested. Error bars represent standard deviation from replicate measurements. C) Left—Table summarizing the obtained k values (µM min^−1^) for the tested reactions. Right—Graphical representation of the % comparison of the k values in the absence and in the presence of ADPr, compound **3** and GS‐441,524 for Nsp3b and MERS‐CoV macrodomain.

To evaluate the inhibitory efficacy of compound **3** on the Nsp3b and MERS‐CoV macrodomains, we applied a previously established de‐MARylation assay with mono‐ADP‐ribosylated GST‐hPARP10ADP‐ribosyl transferase domain (hPARP10 ART) as the substrate (**Figure** **5**).^[^ ^10^ ^]^ The initial substrate decay rate constant (k), which indicates the rate of ADP‐ribose removal from the substrate, was determined in the absence and/or presence of ADPr, GS‐441524, and compound **3** at a molar ratio of 1:100. A low k value corresponds to a potent inhibition of macrodomain hydrolase activity.

In the absence of an inhibitor, the free Nsp3B demonstrated activity with a k value of 0.108 ± 0.004 µM min^–^^1^. ADPr, the physiological ligand, was used as a benchmark at a 1:100 molar ratio, resulting in a 45% reduction of the k value (0.059 ± 0.003 µM min^–^^1^). In the presence of GS‐441524 and compound **3**, at an equivalent ratio (1:100), the reactions exhibit k values that are 33% and 47% lower, respectively, than the value corresponding to the free form of the macrodomain. Compound **3** displayed a different profile on MERS‐CoV macrodomain.

At a 1:100 ratio, ADPr slowed MERS‐CoV‐MD enzymatic activity 32%, while compound **3** only 16%.

In contrast, compound **3** had a comparatively smaller effect on MERS‐CoV Mac1, reducing the k value by only ≈16%, while ADPr reduced it by ≈32%. This prelimary observation suggests that compound **3** may exhibit some degree of target selectivity. Further investigations involving dose–response analyses and statistical evaluation across multiple replicates will be necessary to substantiate any potential selectivity or differential inhibitory potency.

### 2.7 Differential Binding of Compound 3 to Viral and Host Macrodomains Revealed by Isothermal Titration Calorimetry

**Figure 6:**
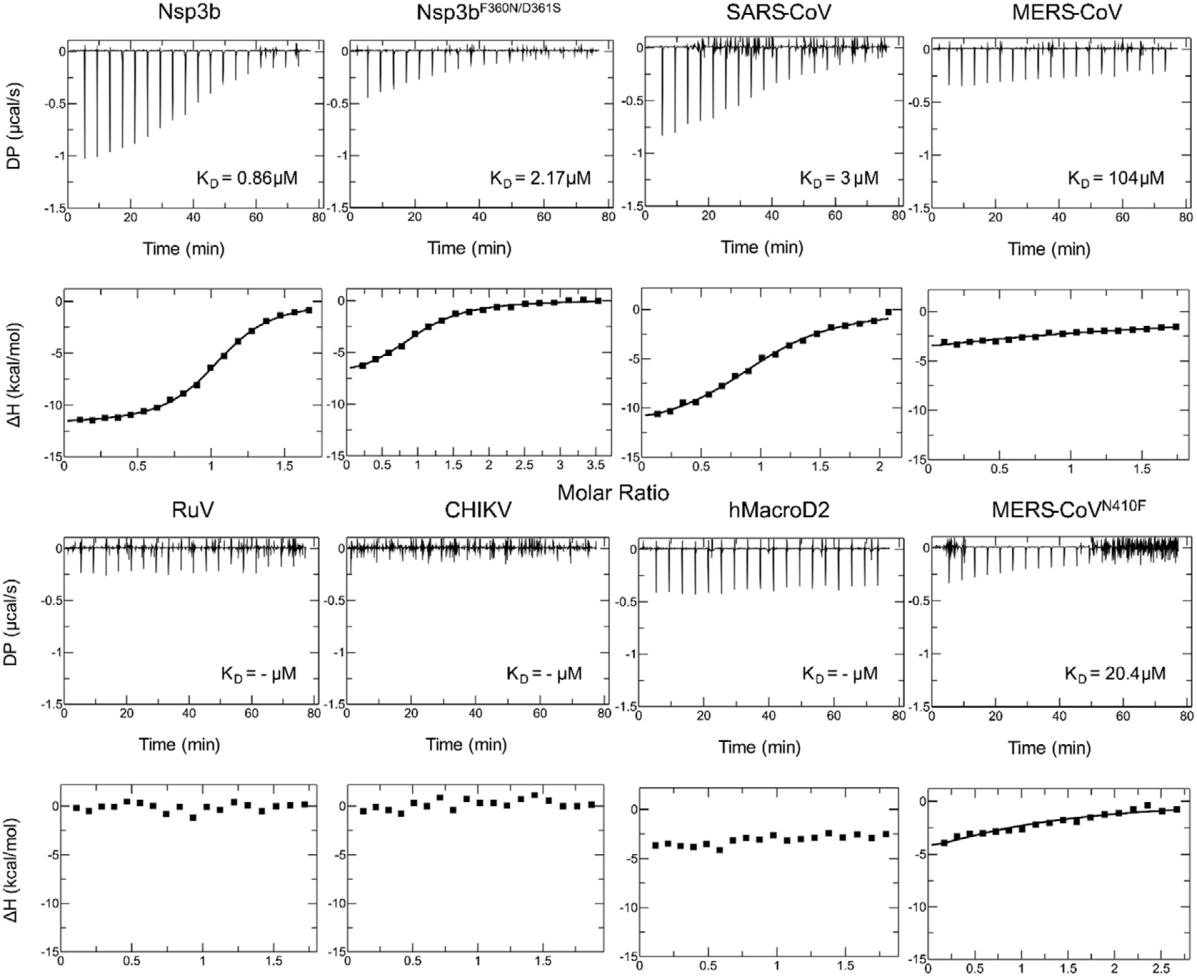
ITC analysis of compound **3** binding to different macrodomain proteins. Each plot's upper graph displays the raw measurement, while the lower graph displays the best fit function (solid line) and intergraded heat per injection (squares). For every interaction, the resulting KD values are reported. **Table** **1** provides a summary of all the thermodynamic parameters.

To characterize the binding selectivity of compound **3**, we conducted ITC against a panel of viral and host macrodomains, including wild‐type and mutant variants. In contrast to the wild‐type (Figure 2B), the SARS‐Cov2 F360N/D361S mutant, residues which were observed in our structure to contribute to ligand binding, exhibited a significantly weaker interaction, with a *K*_D_ of 2.17 ± 0.16 µM (**Figure** **6**). This double mutant was expected to have lower affinity for compound **3** as the F360N and its adjacent residue D361 were also crucial for bind the parent molecule GS‐44124 as shown in our previous study.^[^ ^10^ ^]^ The thermodynamic signature for this mutant revealed a markedly reduced enthalpic gain (ΔH = −7.83 ± 0.15 kcal mol^−1^) and a negligible entropy contribution (TΔS ≈ 0.10 kcal mol^−1^), suggesting that the introduced mutations disrupt key hydrogen bonding interactions critical for high‐affinity binding. Similarly, compound **3** bound to the MERS‐CoV N410F mutant macrodomain with moderate affinity (*K*_D_ = 20.4 ± 0.14 µM) and a weaker enthalpic profile (ΔH = −9.56 ± 0.10 kcal mol^−1^), indicating partial retention of binding determinants (Figure 6). Showcasing that the phenylalanine at this position remains significant also for binding compound **3** as was for GS‐441524. Furthermore, an even lower affinity (*K*_D_ = 104 ± 13 µM) was detected for the wild‐type MERS‐CoV macrodomain, whereas for the host‐derived hMacroD2, the alphavirus CHIKV macrodomain and the rubivirus Rubella virus (RuV) macrodomain, no interaction was observed as evidenced by the flat thermograms and the absence of heat exchange. Notably, the parent compound GS‐441524 showed no significant interaction with CHIKV macrodomain in our previous study.^[^ ^10^ ^]^ Its interaction with RuV macrodomain has not been established, but the absence of the key residue F360, substituted with a proline, argues against high‐affinity binding. The above results confirm that compound **3** lacks measurable affinity for these proteins under the applied experimental conditions.

**Table 1:** Thermodynamic parameters of the interaction between viral, human macrodomains, and compound **3**.

Macrodomain	*K*_D_ [μ*M*]	N	ΔH [kcal mol^–1^]	−TΔS [kcal mol^–1^]	ΔG [kcal mol^–1^]
Nsp3b	0.86 ± 0.04	1.03	−12.0 ± 0.07	3.71	−8.28
Nsp3b^F360N/D361S^	2.17 ± 0.16	1*	−7.83 ± 0.15	0.104	−7.73
SARS‐CoV	3.08 ± 0.33	1*	−12.5 ± 0.3	4.96	−7.52
MERS‐CoV	104 ± 13	1*	N.D.	N.D.	−5.43
MERS‐CoV^N410F^	20.4 ± 0.14	1*	N.D.	N.D.	−6.40
Rubella	N.D.	1*	N.D.	N.D.	N.D.
CHIKV	N.D.	1*	N.D.	N.D.	N.D.
hMacroD2	N.D.	1*	N.D.	N.D.	N.D.

These results demonstrate that compound **3** achieves selective and high‐affinity recognition of the Nsp3b over both viral mutants and host macrodomains. The binding appears to be governed predominantly by enthalpic interactions, with sensitivity to mutations that alter the hydrogen bonding network of the phosphate‐binding pocket.

### 2.8 Antiviral Activity of Compound 3 in Cell‐Based Assays

We next evaluated whether this biochemical selectivity manifests as antiviral activity in cells for SARS‐CoV‐2, as well as for Chikungunya Virus. In VeroE6‐TMPRSS2 cells infected with SARS‐CoV‐2 B.1, compound 3 showed no measurable inhibition up to 40 µM while remaining noncytotoxic (CC_50_ > 40 µM); the internal control remdesivir produced the expected potency (EC_50_ 5.48 µM), validating assay performance. In a CHIKV (OPY1) infection assay in VeroE6 cells, compound 3 displayed modest activity with EC_50_ values of 21.8 µM and CC_50_ > 40 µM, while Favipiravir gave EC_50_ = 11.34 µM (Figure S16, Supporting Information). Thus, despite selective, enthalpy‐driven binding to SARS‐CoV‐2 Nsp3b in vitro, compound 3 does not inhibit SARS‐CoV‐2 replication in this cellular context, whereas altering CHIKV replication in the >10 µM range. The disconnect between ITC and antiviral readouts may reflect cellular liabilities such as limited intracellular exposure, efflux, or metabolism or biology of the target, including context‐dependent non‐essentiality of macrodomain inhibition for replication in vitro. The favorable cytotoxicity margin nonetheless supports further optimization. Full dose–response curves, and viability controls are provided in the Supporting Information Figure S16.

## 3 Conclusion

We report the structure‐guided design of compound **3**, a sulfamoyl‐modified analog of GS‐441524, as a potent and selective inhibitor of the SARS‐CoV‐2 Nsp3b. This work provides compelling evidence that nonphosphorylated phosphate mimetics—specifically sulfamoyl groups—are viable and effective alternatives to classical phosphate‐based ligands for targeting viral Nsp3b.^[^ ^10^ ^]^ The strategic replacement of the 5′‐phosphate with a sulfamoyl group was driven by the dual aim of preserving essential molecular recognition within the phosphate‐binding pocket while exploring chemical modifications that may favor improved molecular properties relevant for future optimization.

Compound **3** demonstrated a significant enhancement in binding affinity, exhibiting a dissociation constant (K_D_) of 0.86 µM, as determined by ITC, a nearly tenfold improvement over GS‐441524 (K_D_ of 10 µM) and a substantial enhancement over ADPr (K_D_ of 6 µM). The binding thermodynamics were enthalpy‐dominated (ΔH = −12.00 kcal mol^−1^), suggesting a network of strong and specific interactions. NMR‐based structural analysis revealed that the sulfamoyl moiety engages the conserved phosphate‐binding subsite through a triad of hydrogen bonds with the backbone amides of Val253 and Ile335 and the carbonyl oxygen of Gly334. Additionally, the potential for a cation–*π* interaction between the protonated sulfonamide nitrogen (pK_a_ = 9.37) and the aromatic ring of Phe336 provides a unique stabilizing interaction that is inaccessible to negatively charged phosphate groups. Nevertheless, we note that this high binding affinity does not directly translate into equally strong inhibition of deMARylation activity, indicating that recognition and catalytic inhibition are not fully coupled. This limitation provides valuable guidance for further scaffold optimization.

Our work builds directly on recent efforts to target viral macrodomains. Alhammed et al. established the importance of macrodomain function in immune evasion and viral pathogenicity, highlighting the therapeutic relevance of Nsp3b despite its nonessentiality for replication.^[^ ^5^ ^]^ O’Connor et al. introduced ADPr analogs and ProTide derivatives.^[^ ^6^ ^]^ Their ligand lacked structural characterization in the viral context. Schuller et al. developed phosphoaramidate‐based nanomolar inhibitors but acknowledged challenges associated with their synthetic complexity.^[^ ^2^ ^]^ Most reported Nsp3b inhibitors to date have relied on ADPr derivatives or phosphate‐containing analogs, these molecules often suffer from poor drug‐like properties, high polarity, and metabolic instability. Non‐nucleotide inhibitors, including sulfonamides and benzoxazoles, have been explored as alternatives, but typically lack structural validation or demonstrate insufficient selectivity for viral macrodomains.^[^ ^18^ ^]^ More recently, additional scaffolds have been reported, such as the triazole‐based inhibitors described by Lee et al. and the fragment‐derived series from Fraser and Correy, both of which highlight promising non‐nucleotide chemotypes that broaden the available chemical space.^[^ ^19^ ^,^ ^20^ ^]^ Likewise, virtual screening campaigns have identified compounds such as folic acid and telmisartan from FDA‐approved drug libraries as potential hits, yet none have progressed to validated lead candidates or demonstrated meaningful target engagement in vitro.^[^ ^18^ ^]^ Earlier analogs including *β*‐O‐methyl‐ADPr (IC_50_ = 127 µM) and *α*‐azido‐ADPr (IC_50_ = 0.49 µM) showed moderate improvements in potency but were limited by synthetic complexity.^[^ ^21^ ^]^ While recent nanomolar inhibitors based on phosphoramidite chemistry (e.g., compound 11, IC_50_ = 30 nM) demonstrate superior potency, their structural complexity may limit translational potential.^[^ ^21^ ^]^ In contrast, compound **3** shows strong binding, selectivity for viral targets, and a well characterized structure, while also being easier to synthesize. Making it promising starting point for future antiviral development.

Efforts to crystallize the **3**•Nsp3b complex were unsuccessful. To overcome this, we employed a hybrid structural strategy integrating solution‐state NMR data with high‐resolution X‐ray crystallography enabled detailed modeling of the binding interface. Specifically, the 1.45 Å structure of Nsp3b in complex with AMP (PDB ID: 6W6Y) served as the docking template for compound **3**, guided by intermolecular distance restraints obtained from X‐filtered NOESY experiments. Among several candidate structures, 6W6Y yielded the most consistent fit, minimizing NOE restraint violations and aligning best with experimental 3_JNH, H*α*_ scalar coupling data—particularly in regions predicted to interact with the sulfamoyl moiety. This integrative approach provided a time‐efficient alternative to de novo structure determination, requiring only a focused set of intermolecular NOEs rather than complete resonance assignment, and could be further accelerated using the NMR molecular replacement approach,^[^ ^16^ ^]^ which enables structure determination even in the absence of chemical shift assignments. Furthermore, when chemical shift assignments of the apo protein are available, they can be rapidly transferred to the ligand‐bound state, thereby minimizing ambiguity and reducing the overall spectroscopic workload. Together, this strategy offers a robust and scalable framework for elucidating high‐resolution protein–ligand complexes when crystallographic data are incomplete or unavailable.

Functionally, compound **3** inhibited macrodomain‐catalyzed de‐MARylation with high selectivity for Nsp3b (SARS‐CoV‐2) over MERS‐CoV and didn’t bind to other selected viral and host macrodomains, except the one of the closely related SARS‐CoV. Consistent with our experiments and the literature, the parent nucleoside GS‐441524 does not bind detectably to RuV or CHIKV macrodomains. Notably, no binding was observed for human MacroD2, underscoring the selectivity potential of this scaffold and its minimal off‐target liability in host systems. However, its reduced activity against other coronavirus macrodomains shows that it is not yet a broad‐spectrum inhibitor, and further SAR efforts will be needed to broaden its utility across coronaviruses.

Selectivity remains a critical challenge in the development of Nsp3b inhibitors, given the structural conservation of the ADPr binding pocket across viral and host macrodomains. Our study identifies several structural features that contribute to the enhanced selectivity of compound **3**. First, the sulfamoyl group serves as a phosphate bioisostere with dual‐functionality—retaining essential hydrogen bond acceptors while uniquely contributing an H‐bond donor (–NH_2_), allowing for interactions (e.g., with G334) that are inaccessible to negatively charged phosphate groups. Second, the protonated state of the sulfonamide enables a cation–*π* interaction with F336. Third, the compact structure of compound **3** avoids steric clashes with the glycine‐rich loop, a limitation commonly observed for bulkier nucleotide analogs.^[^ ^22^ ^]^

From a design standpoint, these features highlight the importance of incorporating nonclassical functional groups that enable new interaction modes beyond those of ADPr. The use of neutral or conditionally charged groups (like sulfamoyl) may also reduce off‐target binding to host enzymes that rely heavily on electrostatic phosphate recognition. Moving forward, further enhancing selectivity may involve targeting secondary subpockets or dynamic loops unique to viral macrodomains, as well as leveraging fragment‐based designs anchored on validated scaffolds such as compound **3**.

Importantly, initial cell‐based infection assays indicated that the biochemical selectivity of compound 3 did not translate into antiviral efficacy against SARS‐CoV‐2 in VeroE6‐TMPRSS2 cells up to 40 µM (EC_50_ > 40 µM; CC_50_ > 40 µM), whereas modest inhibition was observed against CHIKV in VeroE6 cells (EC_50 _ ≈ 21.8 µM; CC_50_ > 40 µM), with remdesivir (EC_50 ≈ 5.48 µM) and favipiravir (EC_50_ ≈ 11.34 µM) serving as positive controls. These findings delineate a gap between strong, enthalpy‐driven target engagement and limited cellular efficacy, pointing to permeability, efflux, metabolic stability, or context‐dependent target essentiality as key areas for optimization.

Taken together, these results establish compound **3** as a validated chemical probe for viral macrodomains and a promising lead for therapeutic development. The use of sulfamoyl phosphate bioisosteres offers a promising approach to mimic phosphate‐based recognition while enabling chemical modifications that may support improved binding characteristics and synthetic accessibility. This strategy may aid the development of next‐generationantivirals targeting viral macrodomains involved in immune evasion and pathogenesis.

## Supporting Information

The authors have cited additional references within the Supporting Information.

## Conflict of Interest

The authors declare no conflict of interest.

## Supporting information

Supplementary Material

## Data Availability

Chemical shift assignment and spatial structure of Nsp3b in complex with compound 3 are available in BMRB and PDB databases under the access codes 35002 and 9RME, respectively.
