# Supplementary material for: Structure‐Based Rational Design of a Selective Hydrolase Inhibitor of the Severe Acute Respiratory Syndrome Coronavirus‐2 Nsp3 Macrodomain
Source: Chembiochem. 2025 Nov 2;26(23):e202500593. doi: 10.1002/cbic.202500593 (PMC12666239; doi:10.1002/cbic.202500593)
Supplement: Supplementary file 1 — Supplementary Material [file CBIC-26-e202500593-s001.pdf]

Supplementary

Structure-based rational design of a selective hydrolase inhibitor of the SARS-CoV-2 Nsp3 macrodomain

Robin Krishnathas,[a] Konstantin Mineev,[a] Nikolaos K. Fourkiotis,[b] Franck Touret,[d] Christos Sideras-Bisdekis,[b] Aikaterini C. Tsika,[b] Santosh Lakshmi Gande,[a] Verena Linhard,[a] Sridhar Sreeramulu,[a] Frank Lennartz,[c] Manfred S. Weiss,[c] Bruno Coutard,[d] Georgios A. Spyroulias,[b] Harald Schwalbe*[a]

[a] Robin Krishnathas, Konstantin Mineev, Santosh Lakshmi Gande, Verena Linhard, Sridhar Sreeamulu
Institute for Organic Chemistry and Chemical Biology, Goethe University Frankfurt, Frankfurt am Main, Germany
Max-von-Laue-Strasse 7, 60438 Frankfurt am Main (Germany)

[b] Nikolaos K. Fourkiotis, Christos Sideras-Bisdekis, Aikaterini C. Tsika, Georgios A. Spyroulias
University of Patras
Department of Pharmacy
26504 Patras, Greece

[c] Frank Lennartz, Manfred S. Weiss
Helmholtz-Zentrum Berlin
Macromolecular Crystallography
Albert-Einstein-Straße 15, 12489 Berlin (Germany)

[d] Franck Touret, Bruno Coutard
Unité des Virus Émergents (UVE)
Aix-Marseille Université,
Università di Corsica, IRD 190, Inserm 1207, IRBA.
Marseille, France

* corresponding authors

Material and Methods

Chemical Reaction

General procedures

All solvents and reagents were used as received, without further purification. Thin-layer chromatography (TLC) was performed using Macherey-Nagel precoated silica gel plates (UV254 fluorescence indicator) and a dichloromethane/methanol eluent system monitored the reaction progress. Flash column chromatography was performed using an Interchim puriFlash XS520Plus system with prepacked Interchim columns.

General reaction procedures

GS-441524 (1.0 equiv.) and pyridine (1.5 equiv.) were dissolved in dry acetonitrile (1.6 mL) under an inert atmosphere. The reaction mixture was cooled to –5 °C, and phosphoryl chloride (5 equiv.) was added slowly dropwise. The reaction was stirred at –5 °C for 2 h and then was allowed to warm up to room temperature overnight. After completion of the reaction, the solvent was removed under vacuum and the residue was applied to the flash chromatography column. The reaction was quenched by the addition of ice water to induce hydrolysis. Reaction progress and purity were monitored by flash chromatography using a mobile phase of acetonitrile/water (5:95) to yield RMP 21%. ^{1}H NMR (500 MHz, D_2O) δ 8.01 (s, 1H), 7.28 (d, J = 4.7 Hz, 1H), 7.08 (d, J = 4.6 Hz, 1H), 4.86 (d, J = 5.2 Hz, 1H), 4.44 (h, J = 3.0 Hz, 1H), 4.35 (t, J = 4.8 Hz, 1H), 4.06 – 3.94 (m, 2H). ^{13}C NMR (126 MHz, D_2O) δ 148.82, 135.76, 128.38, 116.33, 113.86, 113.30, 109.79, 84.72, 77.43, 75.32, 70.24, 63.84. Mass detected: 370.16 (neg M-H).

A solution of GS-441524 (1.0 equiv.) in acetone (50 mL) was treated with p-toluenesulfonic acid monohydrate (10.0 equiv.) and the mixture was stirred overnight at room temperature. The reaction mixture was ended by addition of saturated aqueous $NaHCO_3$, the compound was extracted using ethyl acetate (3 × 20 mL). The combined organic layers were washed with brine, dried over anhydrous $MgSO_4$, and concentrated to 3–4 mL under reduced pressure. Cyclohexane was added to induce precipitation of the crude product, which was subsequently purified by flash chromatography (0–20% MeOH in DCM) to provide the compound 1 to yield 78%. ^{1}H NMR (600 MHz, DMSO) δ: 7.97-7.94 (m, 3H), 6.93 – 6.87 (m, 2H), 5.36 (d, J = 6.6 Hz, 2H), 5.06 (t, J = 5.7 Hz, 1H), 4.88 (dd, J = 6.6, 3.1 Hz, 1H), 4.30 (td, J = 5.3, 3.1 Hz, 1H), 3.52 (ddt, J = 33.0, 11.5, 5.5 Hz, 2H), 1.63 (s, 3H), 1.36 (s, 3H). ^{13}C NMR (151 MHz, DMSO) δ 155.54, 148.08, 122.50, 116.90, 116.19, 115.38, 110.54, 100.83, 85.38, 83.87, 81.52, 79.92, 60.85, 25.84, 25.07. Mass detected: 330.21 (neg M-H).

In a nitrogen-purged 50 mL flask, compound 1 and NaH (1.5 equiv., 60% dispersion in mineral oil) were suspended in tetrahydrofuran (THF, 8 mL), were heated to 55 °C, and were stirred for 1 h. The mixture was cooled to 0 °C and was stirred for an additional hour. A solution of sulfamoyl chloride (1.1 equiv.) in THF was added dropwise over 30 min at 0 °C. The reaction was stirred for 3 h and monitored by TLC. After quenching with MeOH (2 mL), the solvents were evaporated under reduced pressure. The crude product was purified by flash chromatography (gradient: 100% DCM → 10–15% MeOH in DCM) to afford compound 2 of 56%. ^{1}H NMR (500 MHz, DMSO) δ: 7.96 (m, 3H), 7.62 (s, 2H), 6.92 (d, J = 4.7 Hz, 1H), 6.88 (d, J = 4.7 Hz, 1H), 5.42 (d, J = 6.4 Hz, 1H), 4.95 (dd, J = 6.6, 3.2 Hz, 1H), 4.61 (m, 1H), 4.18 – 4.13 (m, 2H), 1.64 (s, 3H), 1.38 (s, 3H). ^{13}C NMR (126 MHz, DMSO) δ:156.03, 148.67, 122.41, 117.51, 116.27, 110.98, 84.31, 82.69, 81.60, 80.63, 67.72, 55.35, 26.37, 25.59. Mass detected: 409.1 (neg M-H).

To obtain compound 3, compound 2 was dissolved in a 1:1 mixture of trifluoroacetic acid (TFA) and water and stirred at 0 °C for 1 h, followed by warming to room temperature. Upon completion (confirmed by TLC), the solvent was removed under reduced pressure. The residue was purified by flash chromatography to (100% DCM → 10–15% MeOH in DCM) to yield 87% of the final product. ^{1}H NMR (500 MHz, DMSO) δ 7.95-7.91 (m, 3H), 7.63 (s, 2H), 6.90 – 6.80 (m, 2H), 6.40 (d, J = 5.8 Hz, 1H), 5.47 (dd, J = 5.8, 2.6 Hz, 1H), 4.26 (ddd, J = 8.6, 6.2, 2.6 Hz, 1H), 3.93 – 3.92 (m, 2H). ^{13}C NMR (126 MHz, DMSO) δ 156.04, 148.40, 123.78, 117.30, 117.04, 110.91, 110.81, 101.40, 81.52, 74.35, 70.52, 68.50, 49.07, 46.25. Mass detected 369.32 (neg M-H).

Protein Expression and Purification

Construct Design. This SARS-CoV-2 reference genome (NCBI accession NC 045512.2), which is identical to the GenBank entry MN908947.3, was used in this study. An Nsp3b construct containing the amino acids V207 to K376 of the full-length Nsp3 protein, was selected based on previous research [1, 3]. This region was cloned into a pET28a(+) expression vector, which includes an N-terminal His_6-tag and a tobacco etch virus (TEV) protease cleavage site, as described previously. As a result, the final protein was obtained with three additional N-terminal residues (Gly-2, His-1, Met0) preceding the native sequence. For the hMacroD2 the residues 7 to 243 of the full-length (fl) hMacroD2 (UniProt entry: A1Z1Q3) were used as the coding sequence. The sequence was purchased from GenScript, already cloned into a pET28a(+) vector, between the restriction sites NdeI and XhoI. The

expressed polypeptide after tag cleavage therefore contained a N-terminal His_6-tag and TEV cleavage site, as well. After tag cleavage the studied polypeptide contained three N-terminal residues (Gly-2, His-1, Met0).

The sequences, as well as protein expression and purification protocols for all other MDs used in this study for the ITC and biochemical assays, have been described elsewhere [7, 11, 14, 16].

Sample preparation. To obtain the 13C/15N-labeled Nsp3b protein, the plasmid was transformed in *E. coli* T7 express cells, cultivated in M9 minimal medium. This medium was supplemented with 1 g/L 15NH4Cl (obtained from Cambridge Isotope Laboratories) and 2 g/L $^{13}C_6$-D-glucose (purchased from Eurisotop). Additionally, 50 µg/mL kanamycin was used as a selective agent. When the cultures reached an OD600 of 0.7 they were subjected to a cold shock on ice for 10 min. before protein expression was induced by adding 0.5 mM IPTG. The temperature was reduced to 18 °C and protein was expressed at 120 revolutions per minute for 13 h. The cells were harvested at 4 000 rpm and the pellet was resuspended in lysis buffer (25 mM Tris-HCl pH 8.0, 150 mM NaCl, 5 mM imidazole, and 10 mM 2-mercaptoethanol) supplemented with one protease inhibitor tablet (cOmplete™, Roche, Germany). Lysis was achieved using a Microfluidics M-110P homogenizer, applying 15000 psi pressure for three cycles under constant ice cooling. The resulting lysate was then clarified by centrifugation at 14000 rpm for 45 minutes in a Beckman Avanti JXN-26 centrifuge. The clarified lysate was subjected to a purification process via fast protein liquid chromatography (Äkta Pure, Cytiva), employing a tandem configuration of two 5 mL HisTrap HP columns (GE Healthcare, USA). After binding, the protein was washed with 4% buffer B (equivalent to buffer A supplied with 500 mM imidazole) and was eluted using 100% buffer B. The eluate fractions containing Nsp3b were pooled and were treated with TEV protease overnight at 4° C while dialyzing against buffer A without imidazole (25 mM Tris-HCl, pH 8.0, 150 mM NaCl, 10 mM 2-mercaptoethanol). After digestion, the protease and His6-tag were removed by a second IMAC purification step. The final purification and buffer exchange step was performed using a HiLoad 26/600 Superdex 75 pg gel filtration column (Cytiva) equilibrated in 25 mM Bis-Tris (pH 6.5), 150 mM NaCl, and 3 mM TCEP. Target protein was concentrated in an Amicon stirred cell using a regenerated cellulose ultrafiltration disc with a 10-kDa cutoff to a final concentration of 20 mg/mL.

Similarly, the plasmid encoding hMacroD2 was transformed into E. cloni® EXPRESS BL21(DE3) (Lucigen) *E. coli* cells and cultivated in minimal M9 medium. The culture of 0.5 L M9 medium (40 mM Na_2HPO_4, 22 mM KH_2PO_4, 8 mM NaCl) containing 0.5 g ^{15}N labeled NH_4Cl and 2 g unlabeled D-glucose, 1 mL from a stock solution containing 0.5 mg/mL biotin and 0.5 mg/mL thiamine, 0.5 mL 1 M Mg_2SO_4, 0.15 mL 1 M $CaCl_2$, 1 mL solution Q (40 mM HCl, 50 mg/L $FeCl_2 \cdot 4H_2O$, 184 mg/L $CaCl_2 \cdot 2H_2O$, 64 mg/L H_3BO_3, 18 mg/L $CoCl_2 \cdot 6H_2O$, 4 mg/L $CuCl_2 \cdot 2H_2O$, 340 mg/L $ZnCl_2$, 710 mg/L $Na_2MoO_4 \cdot 2H_2O$, 40 mg/L $MnCl2 \cdot 4H_2O$), and 50 µg/ml kanamycin was inoculated with a preculture that was grown o.n. containing the cells that was transformed with the above mentioned plasmid. The cells were incubated at 37 °C with shaking at 180 rpm and the expression was induced at an O.D. of 0.6-0.8 by 0.5 mM IPTG. The temperature was lowered to 18 °C and the incubation was continued for 14-16 h at 180 rpm.

The hMacroD2 was purified as follows: cells were harvested by centrifugation at 7000 rpm for 10 min and the cell pellet was resuspended in 25 mL lysis buffer containing 10 mM imidazole, 50 mM Tris pH 7, 1 M NaCl, supplemented with 10% glycerol, 2mM DTT, and 10 µL inhibitors (P8849 Sigma). Then, 10 µL of DNaseI (10 mg/mL) were added and the suspension was sonicated (PMisonix®, Sonicator 4000). Following sonication, the lysate was centrifuged at 13000 rpm for 45 min at 4 °C (Thermo Scientific®, Sorvall Lynx 6000). The soluble fraction containing the His_6-tagged hMacroD2 was loaded onto a HistrapTM HP affinity column (Cytiva), previously equilibrated with 0.1 M $NiSO_4 \cdot 7H_2O$ and binding buffer (10 mM imidazole, 50 mM Tris pH 8, 500 mM NaCl). The column was washed using a stepwise gradient of imidazole in binding buffer (10, 20, 40, 100, 200, 400 mM) and the protein was eluted in the 200-400 mM imidazole fractions. With the use of an Amicon® Ultra 15 mL Centrifugal Filter membrane (nominal molecular weight cutoff 10 kDa) the protein was concentrated to a final volume of 5 mL, and buffer exchange performed, from the elution buffer to 50 mM Tris pH 8, 300 mM NaCl, 2mM DTT. 300 µL of TEV protease (1 mg/mL) were added and the mixture was incubated for 16 h at 4 °C. After 16 h, with the use of an Amicon® Ultra 15 mL Centrifugal Filter membrane (nominal molecular weight cutoff 10 kDa) the protein was concentrated to a final volume of 0.5 mL, and as well buffer exchange performed into 50 mM Tris pH 7 20 mM NaCl, 2 mM DTT. The protein was then subjected to cation exchange chromatography using HitrapTM SP (Cytiva) and was eluted with a step gradient of NaCl. The fractions containing pure protein were pooled and purified through size exclusion chromatography (SEC) in ITC buffer (50 mM HEPES pH 7, 50 mM NaCl, 2 mM EDTA) using a Superdex75 Increase 10/300 GL column (Cytiva). The pure fractions were collected, flash-frozen and stored at -80 °C until use. The purified protein was concentrated to a final stock solution of 5 mg/mL.

NMR Measurements

Initial screening experiments were conducted on the 600 MHz Avance Neo NMR spectrometer, using a protein concentration of 100 µM and 200/1000 µM concentrations of the ligand in the NMR buffer (25 mM Tris-Bis pH 6.5, 150 mM NaCl, 3 mM tris(2-carboxyethyl)phosphine (TCEP), and 20 µM DSS) at 283 K. The protein was uniformly labeled with ^{13}C and ^{15}N isotopes. 5% D_2O was added to all NMR samples. ^1H and BEST-TROSY experiments [6] were recorded at each titration point. To assess the K_D values, chemical shift perturbations (CSPs) were measured [20]:

$$\Delta\delta_{HN} = \sqrt{(0.1\Delta\,\delta_N)^2 + (\Delta\delta_H)^2} \qquad (1),$$

where, δ_N δ_H represent the chemical shift changes for the amide nitrogen and proton respectively, for 6 residues with largest overall CSPs and clearly traceable signals under all three conditions. Then, the K_D was determined by solving numerically the following equation:

$$\frac{\mathrm{CSP(200)}}{\mathrm{CSP(1000)}} = \frac{\mathrm{K_D+3Prot-\sqrt{-8Prot^2+(K_D+3Prot)^2}}}{\mathrm{K_D+11Prot-\sqrt{-40Prot^2+(K_D+1Prot)^2}}} \quad (2),$$

where CSP(200) and CSP(1000) are the CSPs at 200 and 1000 µM concentrations of the ligand, Prot is the total protein concentration, and K_D is the dissociation constant. The resulting K_D magnitudes were averaged to obtain a final value.

The fine titrations were performed for the compounds that have shown or were suspected to demonstrate strong binding, keeping the protein concentration at either 100 (for RTP and RMP) or 20 (for RSA and RdSA) µM. To find the K_D, the CSP dependence on the ligand concentration was approximated by the equation:

$$\mathrm{CSP} = 0.5\ \mathrm{CSP_{max}}\left(\mathrm{K_D + Lig + Prot} - \sqrt{(\mathrm{K_D + Lig + Prot})^2 - 4\mathrm{LigProt}}\right) \ (3),$$

where Lig and Prot are the concentrations of protein and ligand, and CSPmax is the parameter of fit.

To determine the pKa of compound 3, the pH was varied in the NMR sample by adding either 0.5 M HCl or 0.5 M NaOH. For this experiment, the protein was dissolved in the NMR buffer, additionally containing a mixture of 0.5 mM imidazole, formic acid, and piperazine to have the internal pH sensors, as described [2].

All pH titration experiments were carried out at 298 K on an 800 MHz AVIIIHD spectrometer equipped with a triple resonance TCI cryoprobe. Spectra were recorded across a pH range of 4.48 to 13.01, the dependence of the 5'-CH_2 group on the ambient pH was monitored to determine the pKa of the sulfonamoyl group (Figure S15).

The Henderson-Hasselbach derived function:

$$\delta_{\mathrm{obs}} = \frac{\delta_A 10^{(\mathrm{pK_a-pH})} + \delta_B}{1+10^{(\mathrm{pK_a-pH})}} \quad (4)$$

and the Hill equation:

$$\delta_{obs} = \delta_A - \frac{\delta_A - \delta_B}{1+10^{\mathrm{n(pK_a-pH)}}} \quad (5),$$

where δ_{obs} is the measured chemical shift and δ_A/δ_B are the chemical shifts in the protonated and deprotonated states, respectively, employed to analyze the pH-dependent chemical shift data.

In both models, δ_{obs} is the observed chemical shift where δ_A and δ_B are the chemical shifts of the protonated (acid) and deprotonated (base) forms, respectively; pKa is the acid dissociation constant; pH is the solution pH; and n is the Hill coefficient. Nonlinear least-squares fitting was performed using Origin 2023 (OriginLab).

Solution structure of SARS-CoV-2 Nsp3b and 3 complex

To determine the structure of the complex formed between compound 3 and the Nsp3b, an equimolar amount of ligand was added to the 1 mM solution of $^{13}C/^{15}N$-labeled Nsp3b in 25 mM BisTris, 150 mM of NaCl, 3 mM of TCEP at pH 6.5, as aliquot from a 100 mM stock solution in DMSO-d_6. After addition of 5% D_2O, the solution was inserted into a 5 mm shaped NMR tube. NMR spectra were recorded using Avance III 950 MHz and 800 MHz spectrometers both equipped with triple resonance cryoprobe at 298 K. To assign the NMR chemical shifts, the following 3D NMR spectra were recorded: HNCO, HNCA, HNcoCA, HNcaCO, HNCACB, HcCH-TOCSY and hCCH-TOCSY. BEST-TROSY pulse sequences [6] were used to record the triple resonance experiments, all the spectra were recorded with non-uniform sampling and processed in qMDD software [12]. Aromatic sidechains were assigned based on the (HB)CB(CGCC)H experiment [10] and 13C-NOESY-HSQC. The 3J(NH,Hα)-coupling constants were measured via the quantitative- 3JNH,Hα experiment [17, 18]. 3J couplings were scaled assuming the Hα R1 rate of 10 s-1 [18]. The NMR chemical shifts were deposited to the BMRB database under the access code 35002. Completeness of the assignment is provided in the Table S1.

To obtain intermolecular distance restraints, four isotope-filtered experiments were recorded: 3D $^{15}N,^{13}C$-filtered, ^{13}C-edited NOESY-HSQC, 3D $^{15}N,^{13}C$-filtered (80 ms), ^{15}N-edited NOESY-HSQC, 2D (80 ms) ω1, ω 2-15N,13C-filtered-NOESY (120 ms, to assign the ligand chemical shifts) and 2D ω 2-$^{15}N,^{13}C$-filtered-NOESY (40 and 80 ms) [23]. The structure was calculated using the CYANA 3.98 software [8] with a suitable protein structure (selection criteria discussed below) taken as a rigid template. The library file for the ligand was created using CYLIB [22]. Water molecules were removed from the selected X-ray structure. The X-ray structure was regularized using the standard CYANA procedure. After uploading to the CYANA calculation module, all the protein angles were fixed and the conformation of ligand inside the protein was determined by simulated annealing in the presence of NOE-based distance restraints. From the 100 calculated structures, 10 models with the lowest restraint violations were selected. Hydrogen bond restraints were introduced during the final step of the structure calculations for the contacts that were observed in more than 50% of the obtained set. The final structure were used the PDB ID 6W6Y [13] as a protein template, but PDB IDs 7QG7 [21], 7BF5 [15], 6WEY [19] and 7TWX [4] were also analyzed. To rank the structures based on the NMR data, the 3J(NH,Hα)-coupling constants were back-calculated from the X-ray structures using the ϕ-angles as provided by CYANA and the Karpulus parametrization from [9]. This parametrization was found to at best correspond to the 3J(NH,Hα)-coupling constants measured in 3D HNHA experiment. The mean square deviation of the predicted J-couplings from the experimentally measured ones was

calculated, and the template that provided the minimal deviation for the regions 330-339 and 354-363 (the regions that form hydrogen bonds with the nitrile and sulfamoyl moieties of 3) and maximal number of possible H-bonds were selected for the final analysis.

MARylation/de-MARylation assays, immunoblotting and ITC-measurements

MARylation assays were performed as previously described [16] using as a substrate the GST-hPARP10 ART domain: hPARP10 ART domain was incubated with β-NAD+ (β-NAD+, N1636 Sigma Aldrich) at 1:100 molar ratio for 20 min at 37 °C in 50 mM HEPES pH 7.5, 150 mM NaCl, 0.2 mM DTT, 0.02% IGEPAL® (CA- 630).

To perform the de-MARylation assays, 1 µM MD and 1 µM MARylated substrate were incubated in reaction tubes containing either no additive, or 100-fold ADPr, GS-441524, or compound 3, in a buffer consisting of 50 mM HEPES (pH 7.5), 150 mM NaCl, 0.2 mM DTT, 0.02% IGEPAL® CA-630, and 2% DMSO. For the specified amount of time, each reaction was incubated at 30 °C and 250 rpm. By adding 4x LDS buffer, reactions were halted. The samples separated using a 12% acrylamide Bis-tris gel after being heated for three minutes at 70 °C and centrifuged for five minutes at 14000 rpm and 4 °C. Distinct experiments with n = 3 were carried out.

The separated proteins were transferred to Polyvinylidene fluoride (PVDF) 0.45 µM pore size membrane (Immobilon P-Merck Millipore) using semi-dry Transfer System (BioRad). 5% non-fat dry milk in Tris-Buffered Saline (TBS) pH 7.5 buffer with 0.1% Tween 20 used to block the blot for one h at room temperature. Then, the anti-mono ADPr binding reagent MABE1076 (Merck Millipore), 1:20000, incubated for 14–16 h at 4 °C. Anti-rabbit IgG HRP-linked (7074-Cell Signaling Technology), 1:20 000, was employed as the secondary antibody for the detection. A ChemiDoc Imaging System (BioRad) utilized for viewing, and Image Lab software was employed for quantification.
Quantification of the initial rate of substrate decay (k) performed as previously described [16].

ITC measurements were carried out using a Microcal PEAQ ITC (Malvern, UK) at 25 °C. All components were brought to equilibrium in the ITC buffer comprising 50 mM HEPES pH 7, 50 mM NaCl, and 2 mM EDTA and the appropriate amount of DMSO, to achieve the same concentration in the syringe and the cell. Compound 3 was titrated into 18-26 µM of the different MDs with 19 injections at a concentration of 225 µM. The ITC data were analyzed using the MicroCal PEAQ-ITC Analysis Software (version 1.41) with the fitting model "one set of sites". Protein concentrations were determined with a Q5000 micro-volume spectrophotometer (Quawell) based on the UV absorbance at 280 nm.

***In vitro* antiviral assay**

Cells

Vero E6 cells (ATCC CRL-1586, RRID:CVCL_0574) and VeroE6/TMPRSS2 cells (ID 100978 obtained from CFAR) were cultivated in minimal essential medium (MEM) supplemented with 7.5% heat-inactivated fetal bovine serum (FBS), 1% non-essential amino acids and 1% Penicillin/Streptomycin (all from Life Technologies). VeroE6 TMPRSS2 cells were supplemented with 1% G-418 (Life Technologies. Both cell lines were cultivated under 5% CO2 and at 37.5°C

Virus

SARS-CoV-2 strain BavPat1 (provided by Pr.Christian Drosten through European Virus Archive GLOBAL and chikungunya virus strain Opy1 (La Réunion Island LR2006_OPY1; EVAg 001v-EVA83) were used in this study. Experiments with infectious virus were performed in a biosafety level 3 laboratory.

***In vitro* determination of EC_{50} and CC_{50}**

One day prior the experiment, 96-well culture plates were seeded with 5×10^4 Vero E6 or VeoE6 TMPRSS2 cells in 100µL assay medium per well (containing 2.5% FCS). The next day, eight 3-fold serial dilutions of compound13 or Remdesivir (positive antiviral control from BLDpharm) or Favipiravir (positive antiviral control from Ambeed) were added to the cells using D300e dispenser (TECAN). For the determination of the 50% effective concentrations (four "virus control" wells were supplemented with 25µL of assay medium without any compounds. After 15min, virus diluted in 25µL of assay medium was added to the wells. This quantity of virus was calibrated so that the viral replication was still in the exponential growth phase for the readout, as previously described [5]. Four "cell control" wells were supplemented with 50 µL of assay medium without any compounds or virus. Plates were incubated for 2 days at 37°C prior to quantification of the viral genome by RT-qPCR. Briefly 100 µL of cell supernatant were extracted using QIAamp 96 DNA kit and Qiacube HT robot (both from Qiagen). Viral RNA was quantified by real-time RT-qPCR (GoTaq 1 step RT-qPCR kit, Promega). Quantification was provided by serial dilutions of an appropriate T7-generated synthetic RNA standard. RT-qPCR reactions were performed on QuantStudio 12K Flex Real-Time PCR System (Applied Biosystems) and analyzed using QuantStudio 12K Flex Applied Biosystems software v1.2.3. For the determination of the 50% cytotoxic concentrations (CC_{50}; compound concentration required to reduce by 50% cell viability), the same culture conditions were used, without addition of

the virus, and cell viability was measured using CellTiter Blue® (Promega) following manufacturer's instructions. EC_{50} and CC_{50} were determined using a nonlinear regression (log(inhibitor) vs. response --Variable slope (four parameters) performed on GraphPad Prism 9.

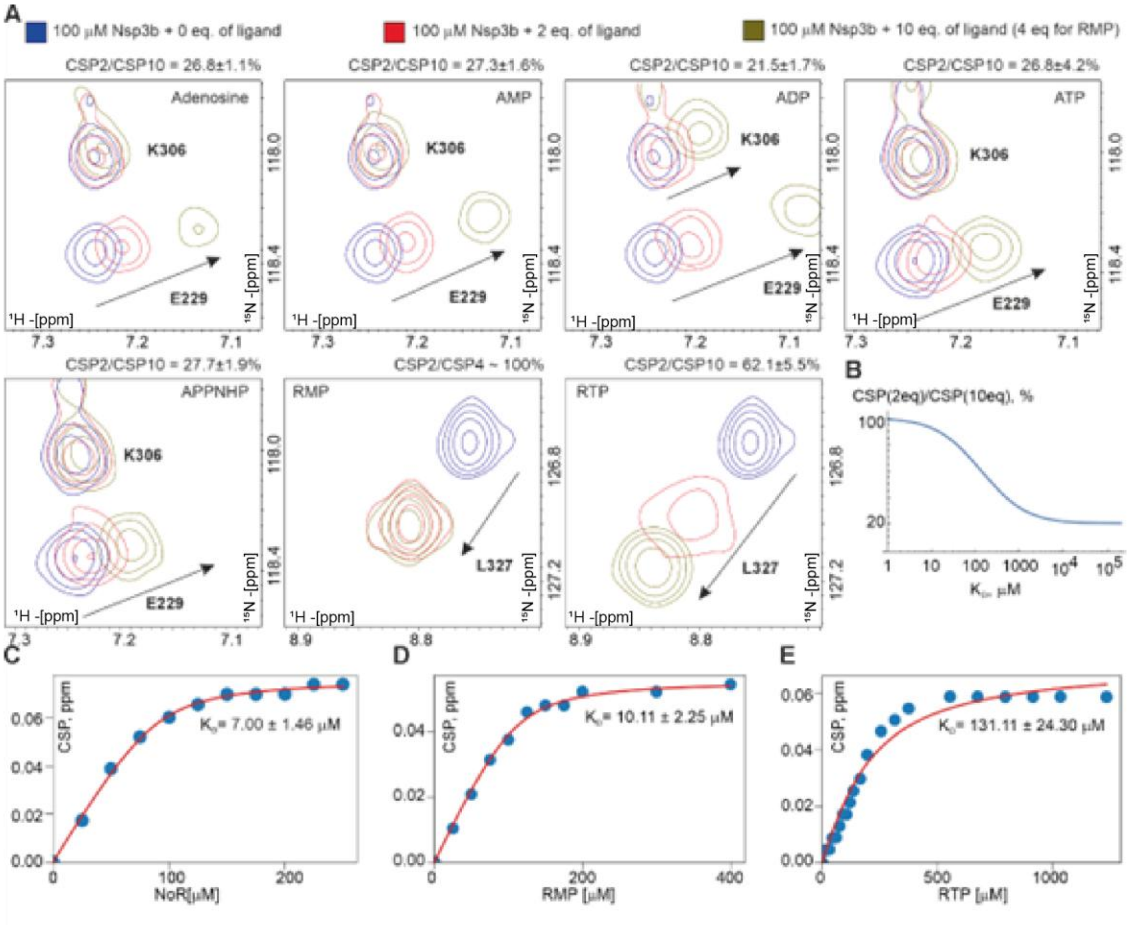

Figure S 1 A – fragments of 2D ^{1}H,^{15}N-HSQC spectra of Nsp3b obtained in the presence of 0 (blue), 2 (red) or 10 (green) equivalents of the corresponding compound at 283K, pH 6.5. Assignment of signals and average ratio of binding-induced CSPs at 2 and 10 equivalents are indicated. B – dependence of the average ratio of binding-induced CSPs at 2 and 10 equivalents of the respective ligand on the binding affinity, K_D. C, D, E – the dependence of CSP (amide groups of the residue for C and D and for E) on the concentration of NoR, RMP and RTP as measured in the titration experiments

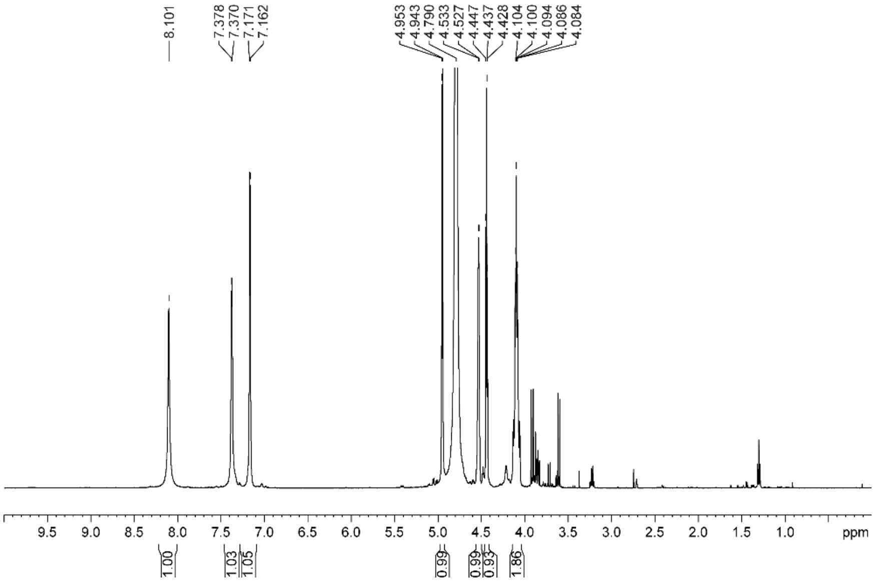

Figure S 2 ^{1}D ^{1}H spectrum of RMP

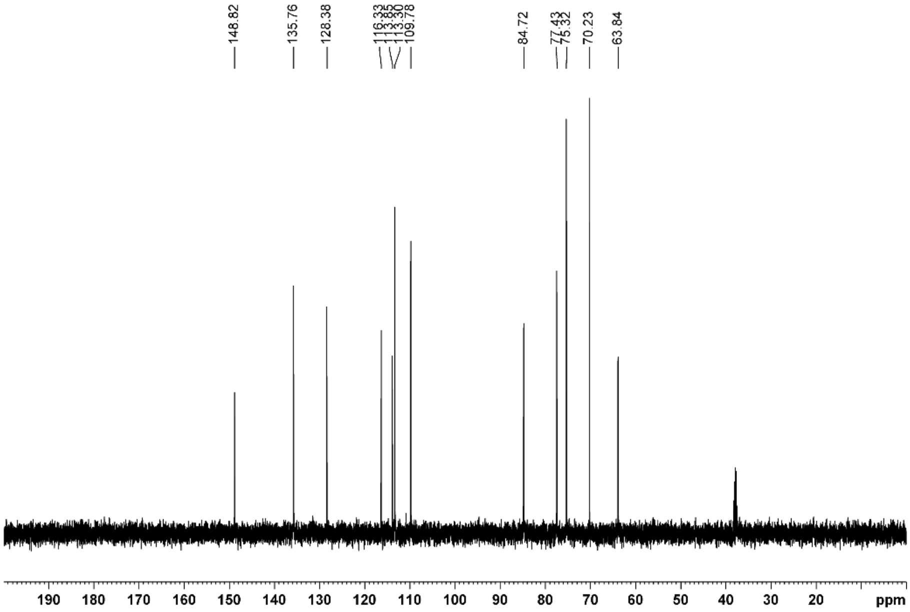

Figure S 3 ^{1}D ^{13}C spectrum of RMP

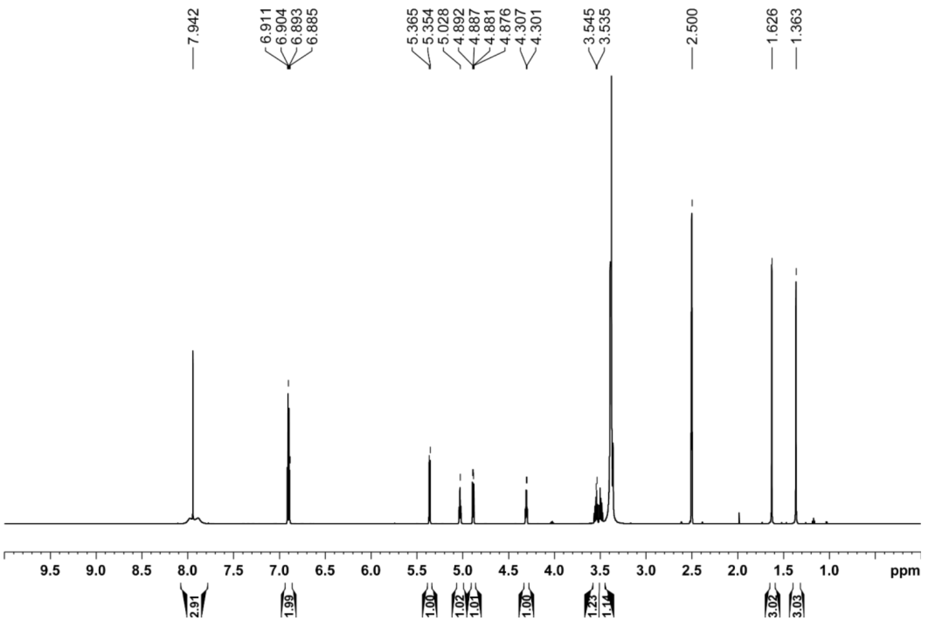

Figure S 4 1D 1H spectrum of compound 1

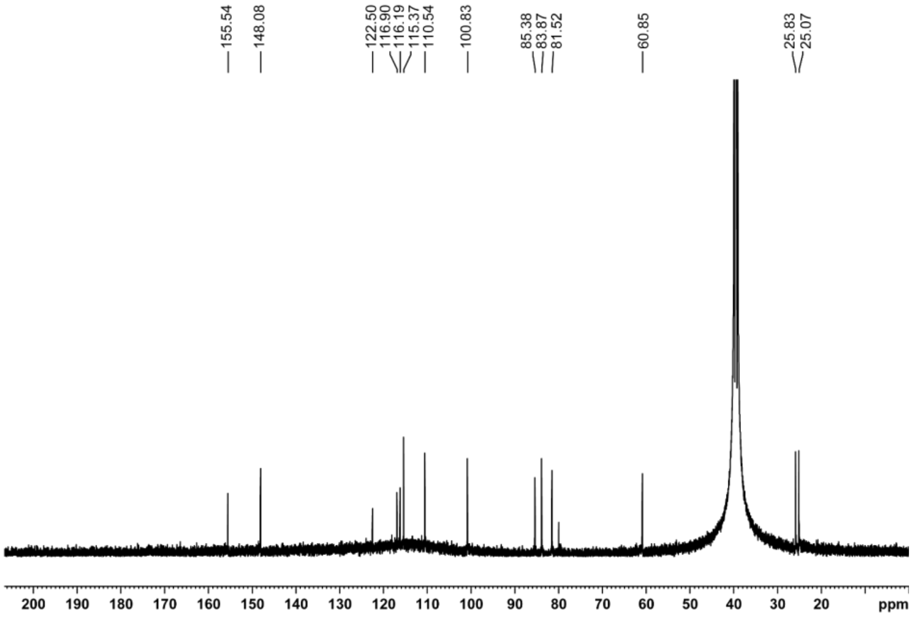

Figure S 5 1D 13C spectrum of compound 1

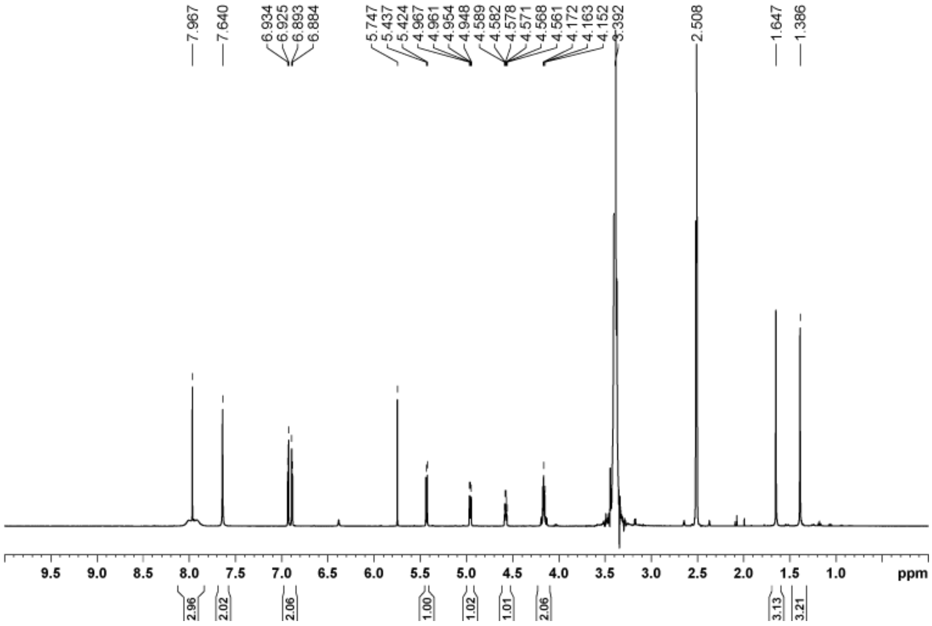

Figure S 6 1D ^{1}H spectrum of compound 2

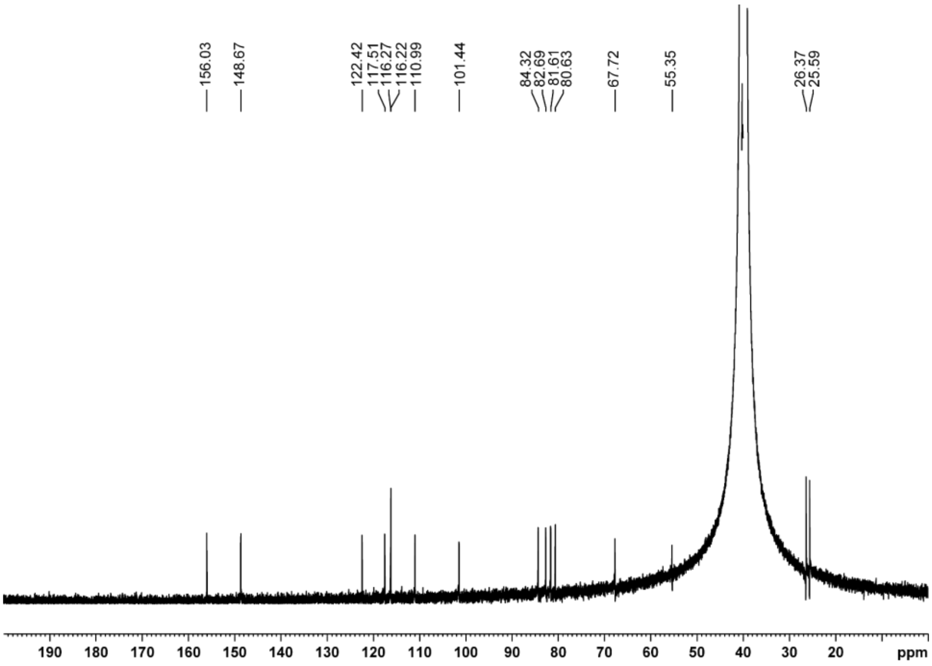

Figure S 7 1D ^{13}C spectra of compound 2

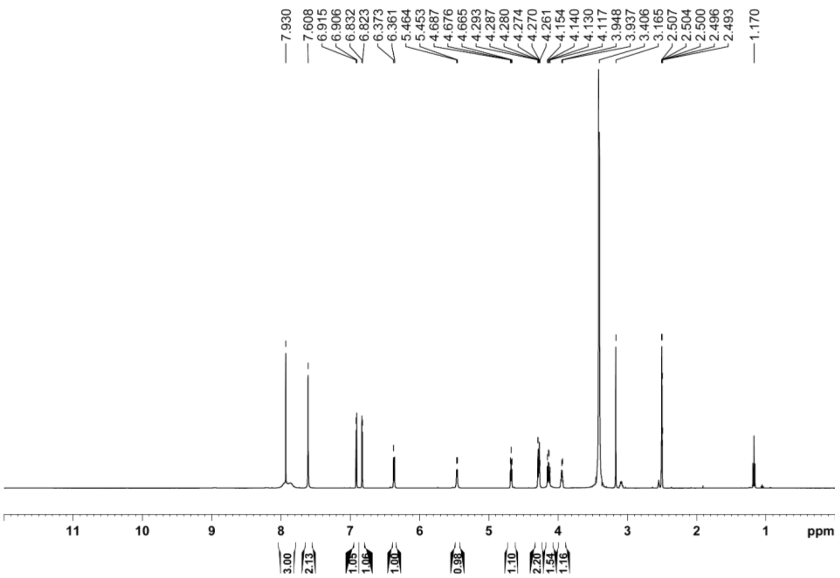

Figure S 8 ^{1}D ^{1}H spectrum of compound **3**

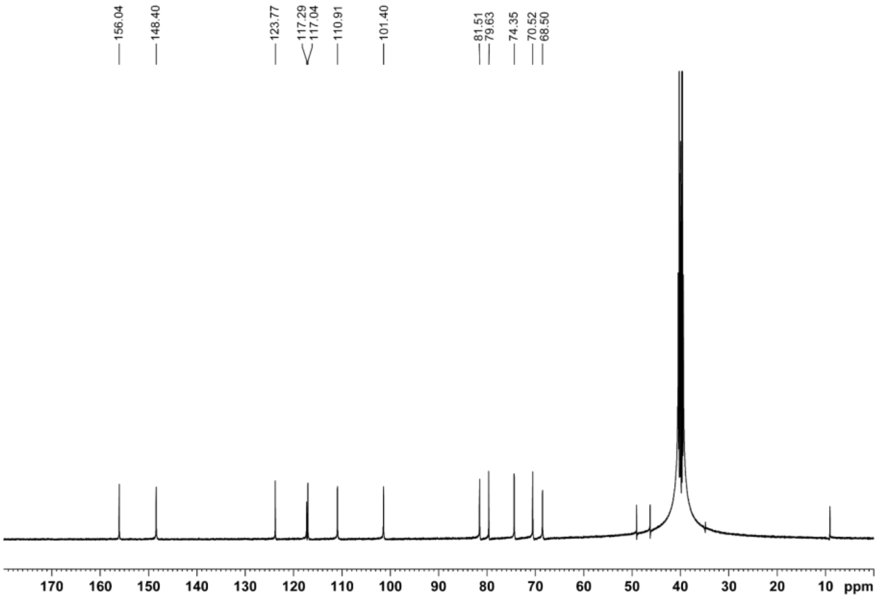

Figure S 9 ^{1}D ^{13}C Spectrum of compound **3**

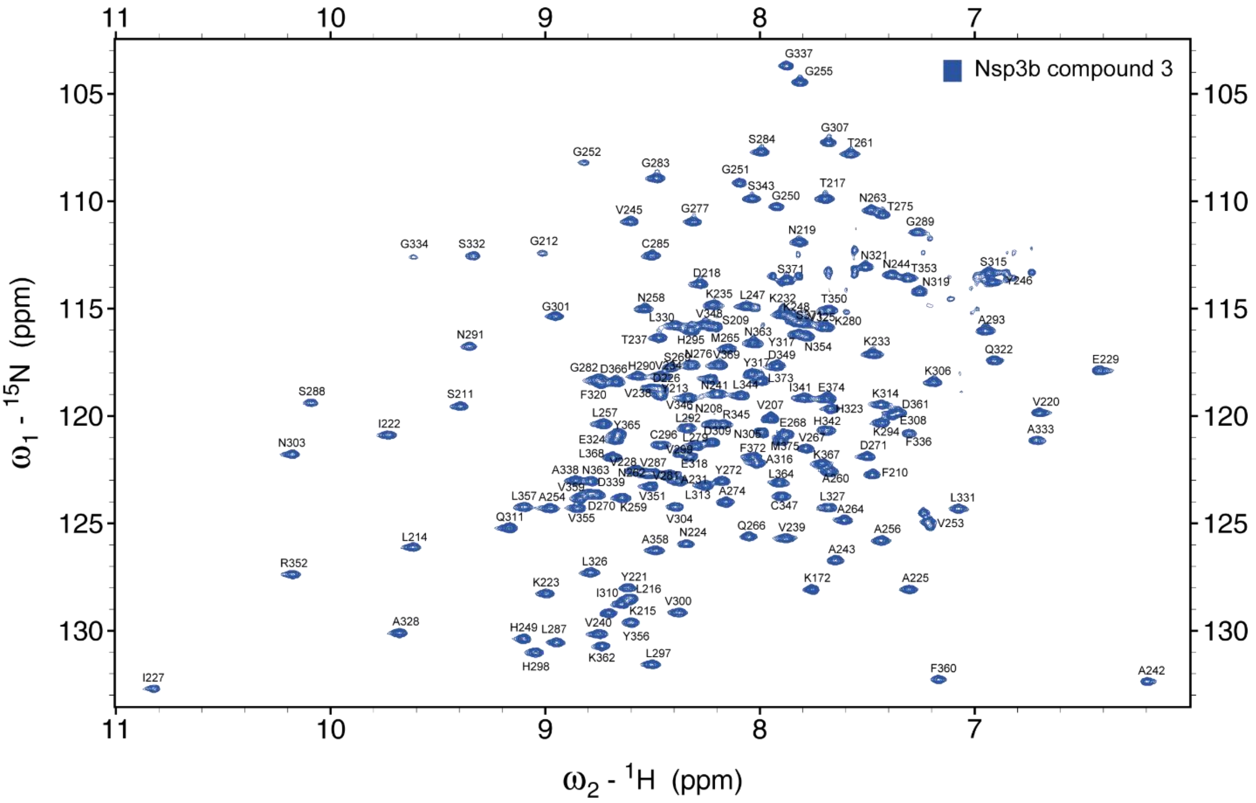

Figure S 10 2D ^{1}H, ^{15}N-TROSY spectrum of Nsp3b at a concentration of 0.9 mM in the presence of 1.8mM of **3,** 25 mM Bis-Tris, pH 6.5, 150 mM NaCl. 5% D_2O, 0.025 mM DSS, 298 K (25 °C), [800 MHz; NS 2, TD 2048]. Assignment of the amide group resonances is indicated.

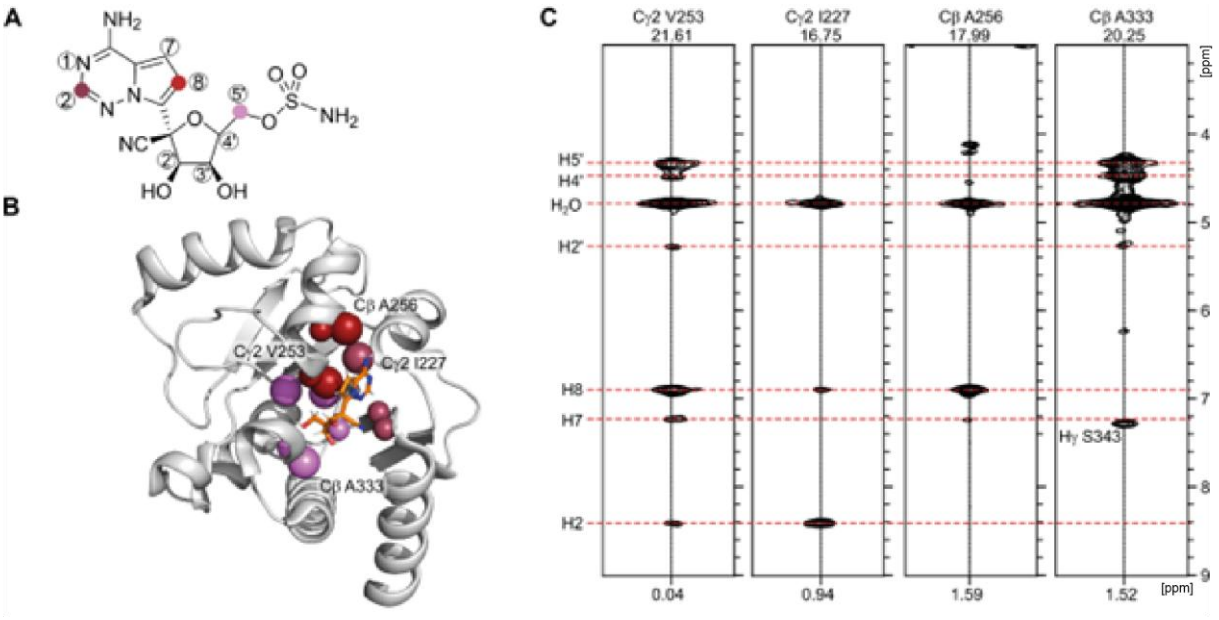

Figure S 11 Intermolecular NOEs detected for 3/Nsp3b complex. A – The structure of 3 is shown with the indication of atom numbering. B – atoms of Nsp3b, involved in the intermolecular NOE contacts with the ligand are shown by spheres on the spatial structure of GS-441524/Nsp3b complex (7QG7). Spheres are painted according to the panel A. C – four representative strips from the 3D $^{13}C/^{15}N$-filtered-^{13}C-edited-NOESY-HSQC spectrum of 3/Nsp3b complex with the indication of signal assignment.

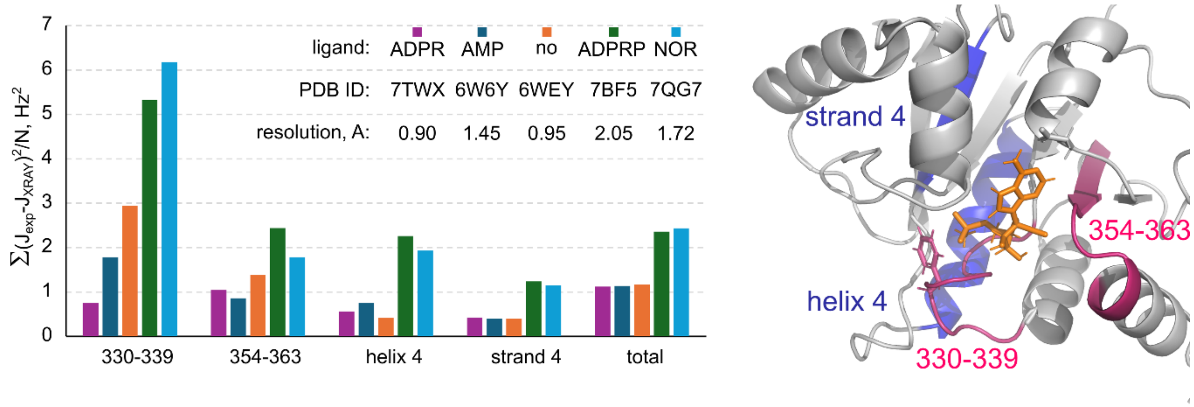

Figure S 12 Selection of the template for the NMR-based structure calculation. The mean squared deviation of predicted and measured $^{3}J_{HNH\alpha}$ couplings for several tested X-ray structures with the PDB IDs indicated. 330-339 and 354-363 (shown by magenta on the macro domain/3 structure) are the protein regions that engage in H-bonds with the nitrile and sulfamoyl groups of the ligand. Helix 4 and strand 4 are reference regions of stable secondary structure, which do not contact with the ligand. Total – the mean square deviation, calculated for all the measured J-couplings.

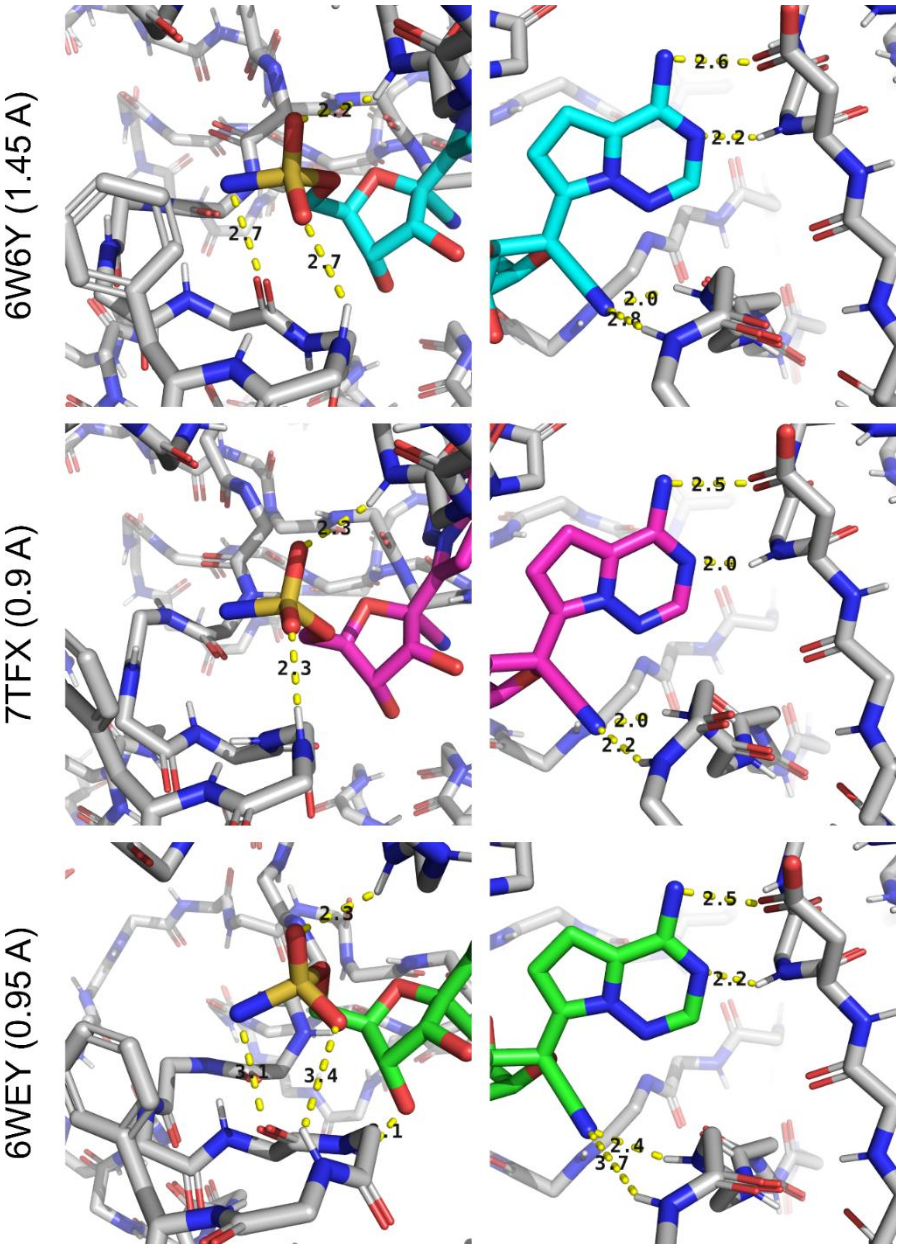

Figure S 13 Effect of the X-ray template on the H-bond network in **3**/Nsp3b complex. Distances corresponding to the possible hydrogen bonding interactions are indicated for the structures that are based on different templates. PDB IDs and resolution of the templates are indicated on the left). 6W6Y reveals 7 hydrogen bonds, 7TFX reveals 6 hydrogen bonds and 6WEY reveals 8 polar interactions, however two of them are too long for the hydrogen bonds. Shown are the templates which at best fit to the NMR-derived J-couplings.

Table S1: Statistics for the NMR data and obtained spatial structures of 3/Nsp3b complex

**Template:**	**6W6Y**	**7TFX**	**7QG7**	**7BF5**	**6WEY**
**Ligand:**	AMP	ADPr	NOR	ADPRP	no ligand
**NMR assignment:**	Completeness, %				
**Backbone**	99				
**Aliphatic sidechains**	98.3				

**Aromatic sidechains**	95.2				
**NMR restraints:**					
**Intermolecular NOEs**	19				
**$^3J_{NH,H\alpha}$ couplings**	151				
**X-ray Resolution, A:**	1.45	0.90	1.72	2.05	0.95
**CYANA target function, A:**					
**With H-bond restraints:**	1.26	2.20	2.09	1.96	4.23
**Without H-bond restraints:**	1.11	1.91	1.50	1.62	3.25

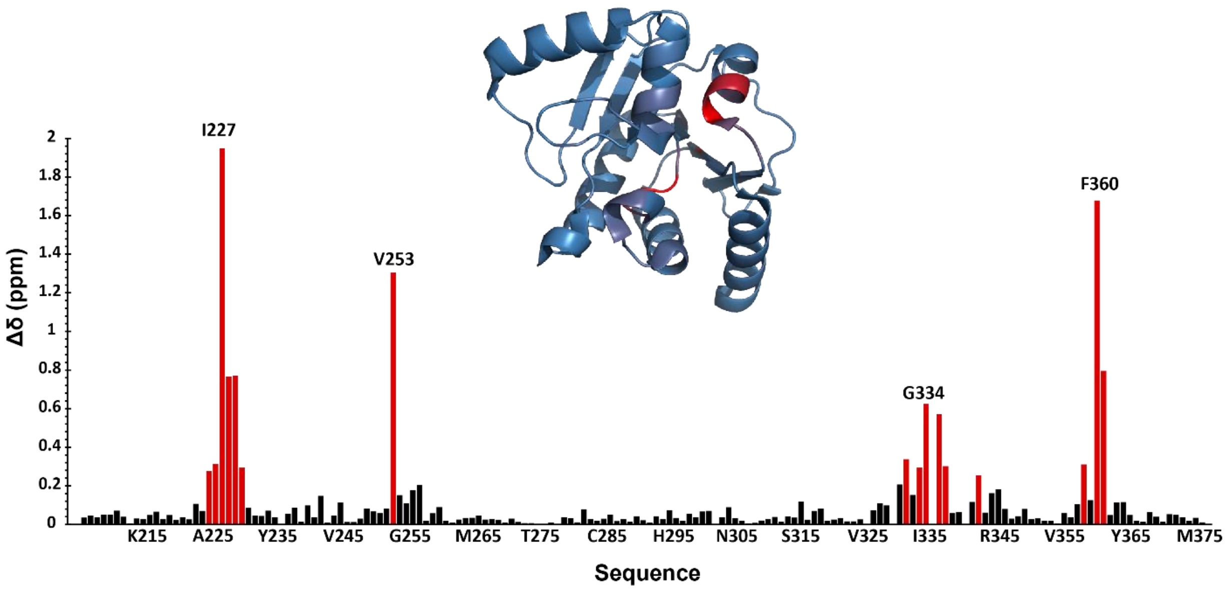

Figure S 14 Chemical shift perturbations induced by the 3 binding to Nsp3b. The bar plot displays the chemical shift perturbations (CSPs) as a function of Nsp3b residue number. Residues exhibiting larger than 0.3 CSPs are highlighted in red.

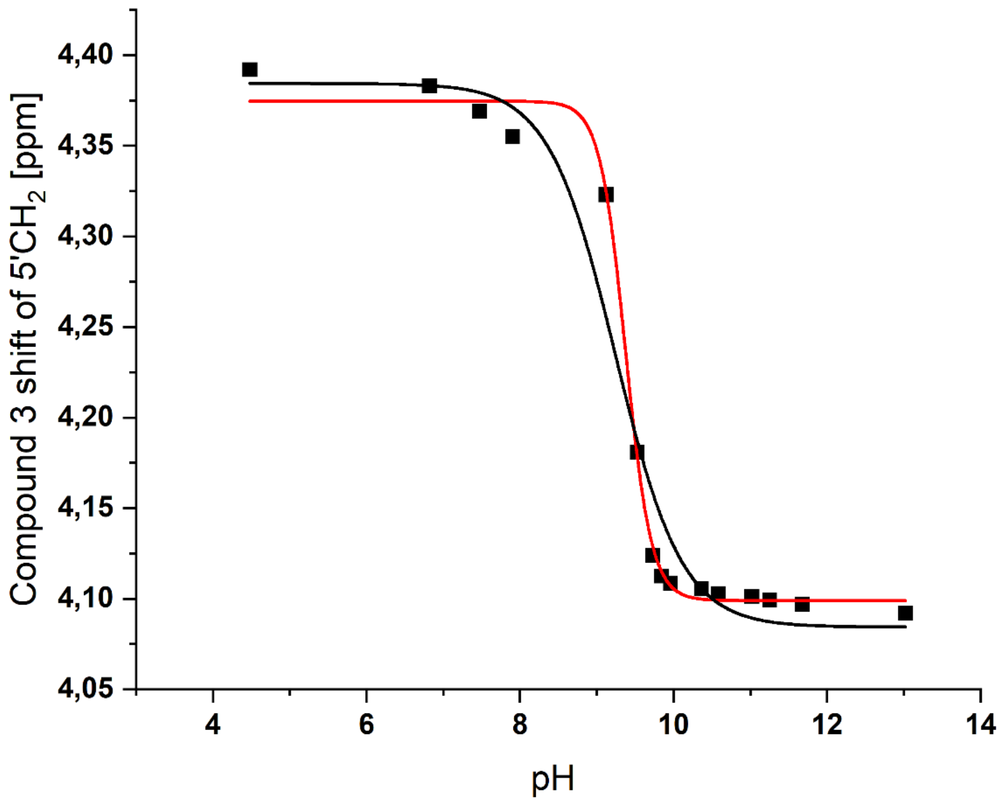

Figure S 15 Determination of the pK_a of the sulfonamoyl group in compound **3**. The chemical shift of the 5′-CH_2 group is plotted as a function of ambient pH. Experimental data points are shown as black squares. The red and black curves represent fits of the data using the Hill equation and a modified Henderson–Hasselbach equation, respectively.

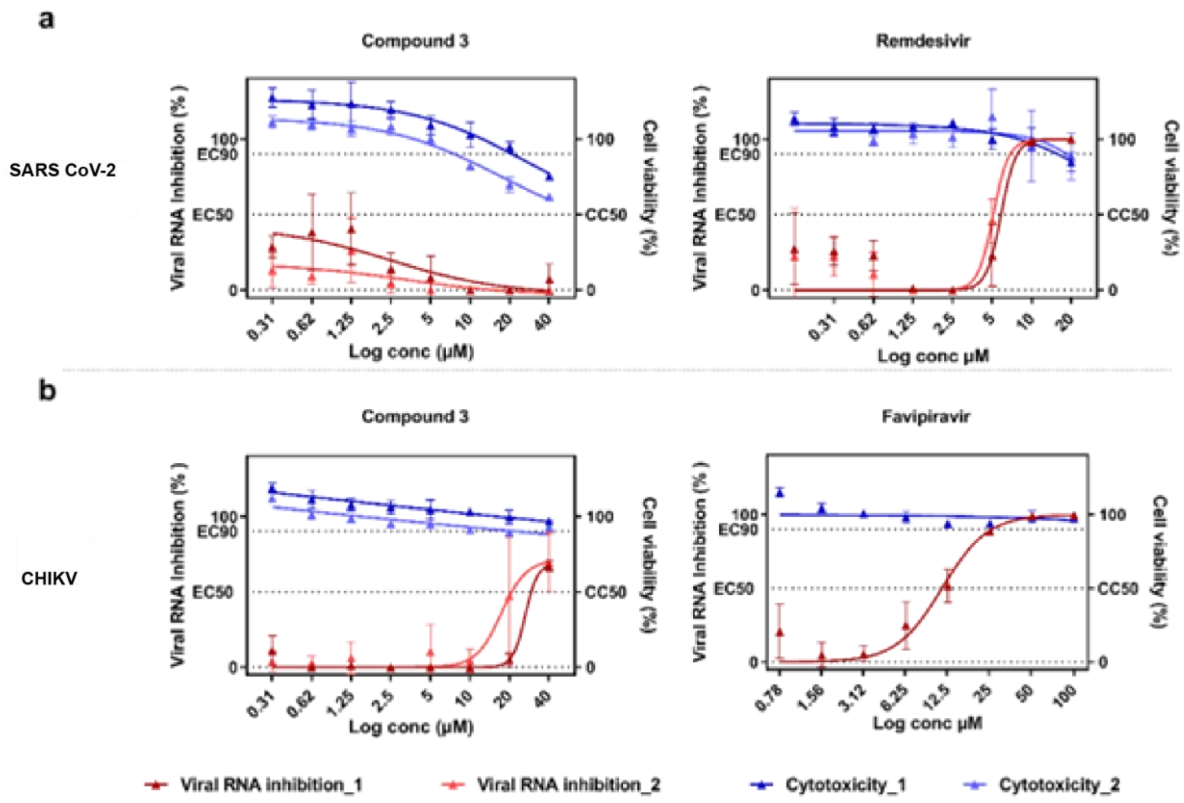

Figure S 16 In vitro evaluation of compound **3** against SARS CoV-2 and chikungunya virus. a) Dose response curves reporting the inhibition activity of compound **3** and Remdesivir against SARS-CoV-2 replication in VeroE6 TMPRSS2 cells b) Dose response curves reporting the inhibition activity of compound **3** and Favipiravir against chikungunya virus replication in VeroE6 cells. Data presented are from three technical replicates, and error bars show mean±s.d.

References

[1] Nadide Altincekic, Sophie M. Korn, Nusrat S. Qureshi, Marie Dujardin, Martí Ninot-Pedrosa, Rupert Abele, Marie J. Abi Saad, Caterina Alfano, Fabio C. L. Almeida, Islam Alshamleh, Gisele C. de Amorim, Thomas K. Anderson, Cristiane D. Anobom, Chelsea Anorma, Jasleen K. Bains, Adriaan Bax, Martin Blackledge, Julius Blechar, Anja Böckmann, Louis Brigandat, Anna Bula, Matthias Bütikofer, Aldo R. Camacho-Zarco, Teresa Carlomagno, Icaro P. Caruso, Betül Ceylan, Apirat Chaikuad, Feixia Chu, Laura Cole, Marquise G. Crosby, Vanessa de Jesus, Karthikeyan Dhamotharan, Isabella C. Felli, Jan Ferner, Yanick Fleischmann, Marie-Laure Fogeron, Nikolaos K. Fourkiotis, Christin Fuks, Boris Fürtig, Angelo Gallo, Santosh L. Gande, Juan A. Gerez, Dhiman Ghosh, Francisco Gomes-Neto, Oksana Gorbatyuk, Serafima Guseva, Carolin Hacker, Sabine Häfner, Bing Hao, Bruno Hargittay, K. Henzler-Wildman, Jeffrey C. Hoch, Katharina F. Hohmann, Marie T. Hutchison, Kristaps Jaudzems, Katarina Jović, Janina Kaderli, Gints Kalniņš, Iveta Kaņepe, Robert N. Kirchdoerfer, John Kirkpatrick, Stefan Knapp, Robin Krishnathas, Felicitas Kutz, Susanne zur Lage, Roderick Lambertz, Andras Lang, Douglas Laurents, Lauriane Lecoq, Verena Linhard, Frank Löhr, Anas Malki, Luiza M. Bessa, Rachel W. Martin, Tobias Matzel, Damien Maurin, Seth W. McNutt, Nathane C. Mebus-Antunes, Beat H. Meier, Nathalie Meiser, Miguel Mompeán, Elisa Monaca, Roland Montserret, Laura Mariño Perez, Celine Moser, Claudia Muhle-Goll, Thais C. Neves-Martins, Xiamonin Ni, Brenna Norton-Baker, Roberta Pierattelli, Letizia Pontoriero, Yulia Pustovalova, Oliver Ohlenschläger, Julien Orts, Andrea T. Da Poian, Dennis J. Pyper, Christian Richter, Roland Riek, Chad M. Rienstra, Angus Robertson, Anderson S. Pinheiro, Raffaele Sabbatella, Nicola Salvi, Krishna Saxena, Linda Schulte, Marco Schiavina, Harald Schwalbe, Mara Silber, Marcius S. Da Almeida, Marc A. Sprague-Piercy, Georgios A. Spyroulias, Sridhar Sreeramulu, Jan-Niklas Tants, Kaspars Tārs, Felix Torres, Sabrina Töws, Miguel Á. Treviño, Sven Trucks, Aikaterini C. Tsika, Krisztina Varga, Ying Wang, Marco E. Weber, Julia E. Weigand, Christoph Wiedemann, Julia Wirmer-Bartoschek, Maria A. Wirtz Martin, Johannes Zehnder, Martin Hengesbach, and Andreas Schlundt. 2021. Large-Scale Recombinant Production of the SARS-CoV-2 Proteome for High-Throughput and Structural Biology Applications. *Frontiers in molecular biosciences* 8, 653148. DOI: https://doi.org/10.3389/fmolb.2021.653148.

[2] Olga K. Baryshnikova, Thomas C. Williams, and Brian D. Sykes. 2008. Internal pH indicators for biomolecular NMR. *Journal of biomolecular NMR* 41, 1, 5–7. DOI: https://doi.org/10.1007/s10858-008-9234-6.

[3] F. Cantini, L. Banci, N. Altincekic, J. K. Bains, K. Dhamotharan, C. Fuks, B. Fürtig, S. L. Gande, B. Hargittay, M. Hengesbach, M. T. Hutchison, S. M. Korn, N. Kubatova, F. Kutz, V. Linhard, F. Löhr, N. Meiser, D. J. Pyper, N. S. Qureshi, C. Richter, K. Saxena, A. Schlundt, H. Schwalbe, S. Sreeramulu, J-N Tants, A. Wacker, J. E. Weigand, J. Wöhnert, A. C. Tsika, N. K. Fourkiotis, and G. A. Spyroulias. 2020. 1H, 13C, and 15N backbone chemical shift assignments of the apo and the ADP-ribose bound forms of the macrodomain of SARS-CoV-2 non-structural protein 3b. *Biomolecular NMR assignments* 14, 2, 339–346. DOI: https://doi.org/10.1007/s12104-020-09973-4.

[4] G. J. Correy and J. S. Fraser. 2022. *Crystal structure of SARS-CoV-2 NSP3 macrodomain in complex with ADP-ribose at pH 7 (P43 crystal form)*.

[5] L. Delang, C. Li, A. Tas, G. Quérat, I. C. Albulescu, T. de Burghgraeve, N. A. S. Guerrero, A. Gigante, G. Piorkowski, E. Decroly, D. Jochmans, B. Canard, E. J. Snijder, M. J. Pérez-Pérez, M. J. van Hemert, B. Coutard, P. Leyssen, and J. Neyts. 2016. The viral capping enzyme nsP1: a novel target for the inhibition of chikungunya virus infection. *Scientific reports* 6, 31819. DOI: https://doi.org/10.1038/srep31819.

[6] Adrien Favier and Bernhard Brutscher. 2011. Recovering lost magnetization: polarization enhancement in biomolecular NMR. *Journal of biomolecular NMR* 49, 1, 9–15. DOI: https://doi.org/10.1007/s10858-010-9461-5.

[7] Angelo Gallo, Aikaterini C. Tsika, Nikolaos K. Fourkiotis, Francesca Cantini, Lucia Banci, Sridhar Sreeramulu, Harald Schwalbe, and Georgios A. Spyroulias. 2021. (1)H,(13)C and (15)N chemical shift assignments of the SUD domains of SARS-CoV-2 non-structural protein 3c: "The SUD-M and SUD-C domains". *Biomolecular NMR assignments* 15, 1, 165–171. DOI: https://doi.org/10.1007/s12104-020-10000-9.

[8] Peter Güntert and Lena Buchner. 2015. Combined automated NOE assignment and structure calculation with CYANA. *Journal of biomolecular NMR* 62, 4, 453–471. DOI: https://doi.org/10.1007/s10858-015-9924-9.

[9] Jin-Shan Hu and Ad Bax. 1997. Determination of φ and χ 1 Angles in Proteins from 13 C− 13 C Three-Bond J Couplings Measured by Three-Dimensional Heteronuclear NMR. How Planar Is the Peptide Bond? *J. Am. Chem. Soc.* 119, 27, 6360–6368. DOI: https://doi.org/10.1021/ja970067v.

[10] Frank Löhr, Robert Hänsel, Vladimir V. Rogov, and Volker Dötsch. 2007. Improved pulse sequences for sequence specific assignment of aromatic proton resonances in proteins. *Journal of biomolecular NMR* 37, 3, 205–224. DOI: https://doi.org/10.1007/s10858-006-9128-4.

[11] Michail V. Lykouras, Aikaterini C. Tsika, Julie Lichière, Nicolas Papageorgiou, Bruno Coutard, Detlef Bentrop, and Georgios A. Spyroulias. 2018. NMR study of non-structural proteins-part III: 1H, 13C, 15N backbone and side-chain resonance assignment of macro domain from Chikungunya virus (CHIKV). *Biomolecular NMR assignments* 12, 1, 31–35. DOI: https://doi.org/10.1007/s12104-017-9775-2.

[12] M. Mayzel, K. Kazimierczuk, and V. Y. Orekhov. 2014. The causality principle in the reconstruction of sparse NMR spectra. *Chemical communications (Cambridge, England)* 50, 64, 8947–8950. DOI: https://doi.org/10.1039/c4cc03047h.

[13] K. Michalska, Y. Kim, R. Jedrzejczak, N. Maltseva, M. Endres, A. Mesecar, and A. Joachimiak. 2020. *Crystal Structure of ADP ribose phosphatase of NSP3 from SARS CoV-2 in complex with AMP*.

[14] Danai Moschidi, Nikolaos K. Fourkiotis, Christos Sideras-Bisdekis, Aikaterini C. Tsika, and Georgios A. Spyroulias. 2025. (1)H, (13)C and (15)N chemical shift assignments of Rubella virus macro domain in the free and in the ADPr bound state. *Biomolecular NMR assignments* 19, 1, 117–125. DOI: https://doi.org/10.1007/s12104-025-10227-4.

[15] X. Ni, S. Knapp, and A. Chaikuad. 2021. *Crystal structure of SARS-CoV-2 macrodomain in complex with ADP-ribose-phosphate (ADP-ribose-2'-phosphate, ADPRP)*.

[16] Aikaterini C. Tsika, Angelo Gallo, Nikolaos K. Fourkiotis, Aikaterini I. Argyriou, Sridhar Sreeramulu, Frank Löhr, Vladimir V. Rogov, Christian Richter, Verena Linhard, Santosh L. Gande, Nadide Altincekic, Robin Krishnathas, Isam Elamri, Harald Schwalbe, Jan Wollenhaupt, Manfred S. Weiss, and Georgios A. Spyroulias. 2022. Binding Adaptation of GS-441524 Diversifies Macro Domains and Downregulates SARS-CoV-2 de-MARylation Capacity. *Journal of molecular biology* 434, 16, 167720. DOI: https://doi.org/10.1016/j.jmb.2022.167720.

[17] G. W. Vuister and A. Bax. 1994. Measurement of four-bond HN-H alpha J-couplings in staphylococcal nuclease. *Journal of biomolecular NMR* 4, 2, 193–200. DOI: https://doi.org/10.1007/BF00175247.

[18] Geerten W. Vuister and Ad Bax. 1993. Quantitative J correlation: a new approach for measuring homonuclear three-bond J(HNH.alpha.) coupling constants in 15N-enriched proteins. *J. Am. Chem. Soc.* 115, 17, 7772–7777. DOI: https://doi.org/10.1021/ja00070a024.

[19] N. Vuksanovic and N. R. Silvaggi. 2020. *High-resolution structure of the SARS-CoV-2 NSP3 Macro X domain*.

[20] Mike P. Williamson. 2013. Using chemical shift perturbation to characterise ligand binding. *Progress in nuclear magnetic resonance spectroscopy* 73, 1–16. DOI: https://doi.org/10.1016/j.pnmrs.2013.02.001.

[21] J. Wollenhaupt, V. Linhard, S. Sreeramulu, M. S. Weiss, and H. Schwalbe. 2021. *SARS-CoV-2 macrodomain Nsp3b bound to the remdesivir nucleoside GS-441524*.

[22] Emel M. Yilmaz and Peter Güntert. 2015. NMR structure calculation for all small molecule ligands and non-standard residues from the PDB Chemical Component Dictionary. *Journal of biomolecular NMR* 63, 1, 21–37. DOI: https://doi.org/10.1007/s10858-015-9959-y.

[23] Catherine Zwahlen, Pascale Legault, Sébastien J. F. Vincent, Jack Greenblatt, Robert Konrat, and Lewis E. Kay. 1997. Methods for Measurement of Intermolecular NOEs by Multinuclear NMR Spectroscopy: Application to a Bacteriophage λ N-Peptide/ boxB RNA Complex. *J. Am. Chem. Soc.* 119, 29, 6711–6721. DOI: https://doi.org/10.1021/ja970224q.
